# Supplementary material for: Impact of co-occurring hearing and visual difficulties in childhood on educational outcomes: a longitudinal cohort study
Source: BMJ Paediatr Open. 2019 Apr 3;3(1):e000389. doi: 10.1136/bmjpo-2018-000389 (PMC6542436; doi:10.1136/bmjpo-2018-000389)
Supplement: Supplementary file 1 [file bmjpo-2018-000389supp001.pdf]

Supplementary Material

Appendix A Hearing and vision assessment

The hearing assessment was carried out by audiologists and specially trained staff, and performed as per the British Society of Audiologists standards(13). Air conduction hearing thresholds were measured bilaterally at 500 Hz, 1kHz, 2kHz, 4kHz and 8kHz, and bone conduction thresholds were measured at 4 kHz for all children and at 1kHz if time permitted. Tympanometry was used to measure middle ear function bilaterally. Tympanograms were categorised according to the Jerger's modified Fieullau-Nikolajsen method(14), with type B tympanograms indicating the presence of otitis media with effusion (OME, 'glue ear').

The visual tests were undertaken by trained orthoptists and overseen by an ophthalmologist. Visual acuity was measured monocularly using the LogMAR test and was taken as the best of presenting (with glasses if worn) and presenting-plus-pinhole. Ocular misalignment was assessed using a simultaneous prism cover test and alternate prism cover test, and strabismus was classified according to the direction (convergent, divergent, vertical or mixed) and whether it was manifest (present in normal viewing with both eyes open), or latent (present only when the eyes were dissociated and/or one covered). Additional information regarding previous visual difficulties was obtained via a questionnaire.

Appendix B Mediating factors

Intelligence quotient (IQ), attention, social cognition and behaviour were selected as potential mediating factors. Information on these domains was collected during a research clinic to which all ALSPAC participants were invited to attend at age 8. IQ was tested using a shortened form of the Wechsler Intelligence Scale for Children, 3rd edition (WISC-III)(31); this comprised five verbal subtests and five performance subtests from which a total age-scaled score was derived. Selective attention was assessed using the Sky Search Test, taken from the Test of Everyday Attention for children(32). The Diagnostic Analysis of Non-Verbal Accuracy (DANVA) faces subtest was used to test social cognition; we used a binary measure comprising the proportion of children with >=7 errors on the facial expressions recognition task(33). Finally, behaviour was assessed using the Strength and Difficulties Questionnaire (SDQ), completed by parents when children were 9 years of age(34). This comprises questions relating to hyperactivity, emotional symptoms, conduct and peer problems.

Appendix C Multiple imputation for missing data

To minimise the potential bias introduced by missing data, we performed multiple imputation using the mi impute command in Stata v.15.0. The exposure, confounding and mediating factors and outcome variables included in the final model were imputed in the sample of 8,205 that attended the research clinic at 7 years and were eligible for inclusion in the study sample. **In addition, we identified factors which predicted missingness using logistic regression analyses and imputed these variables (maternal smoking during pregnancy, male gender and maternal age <25 years). All of these variables were included in the final model, thus the assumption of "missing at random" is supported.**

Table ST1 summarises the variables and proportion of missing values and compares observed distributions to their distributions after the multivariate imputation for used to impute missing values.

Variable	Number missing (%)	Observed data	Complete data
		% for categorical/binary variables or mean (SD) for continuous variables	
**Hearing impairment** (OME and/or conductive hearing loss)	802 (9.8%)	10.3	10.4
**Visual impairment** (Amblyopia and/or strabismus and/or reduced acuity)	1005 (12.2%)	6.9	7.0
**Maternal age <25 years**	451 (5.5%)	21.6	21.8
**Admission to SCBU**	821 (10.0%)	6.7	6.9
**Ethnicity – white**	860 (10.5%)	96.0	95.9
**Maternal smoking during pregnancy**	1410 (17.2%)	20.0	20.3
**Achievement of ≥ Level 4 at KS2 English**	49 (0.6%)	75.2	75.2
**Achievement of ≥ Level 4 at KS2 Maths**	1050 (12.8%)	82.9	81.9
**Achievement of ≥ Level 4 at KS2 Science**	47 (0.6%)	82.9	83.0
**Achievement of ≥ 5 GCSEs at A*- C** (including Maths & English)	1256 (15.3%)	59.8	56.3
**DANVA** (≥7 mistakes)	2038 (24.8%)	22.3	23.3
**Family Adversity Index (1 – 12)**	643 (7.8%)	1.75 (1.89)	1.75 (1.89)
**Wechsler Intelligence Scale (WISC-III)**	1547 (18.9%)	104.7 (16.3)	104.5 (16.4)
**Strengths & Difficulties Questionnaire (SDQ)**	1703 (20.8%)	6.75 (4.84)	6.77 (4.96)
**Sky Search Test**	1710 (20.8%)	5.20 (1.9)	5.21 (2.0)
**Index of Multiple Deprivations**	1968 (24.0%)		
**1**		33.1	32.2
**2**		19.6	19.3
**3**		18.9	18.9
**4**		15.3	15.8
**5**		13.1	13.8
**Parity**	716 (8.7%)		
**0**		46.1	46.3
**1**		36.0	35.8
**>=2**		17.9	17.9

Table ST1: Proportion of data missing for the variables imputed, and distribution of values in the observed data and after imputation

Characteristic	Attended clinic at 7 years (N = 8,283)		Did not attend clinic at 7 years (N = 6,402)		P-value
	N	Percentage of complete data (%)	N	Percentage of complete data (%)	
**Sex**					
Male	4,201	50.7	3,335	52.1	0.09
Female	4,082	49.3	3,066	47.9	
**Low birthweight (<2,500g)**					
Yes	377	4.9	370	6.1	0.00
Missing	549	-	343	-	
**Premature (gestation <37 weeks)**					
Yes	451	5.8	394	6.4	0.11
Missing	455	-	257	-	
**Admission to SCBU**					
Yes	507	6.8	355	7.5	0.17
Missing	830	-	1,644	-	
**Ethnicity**					
White	7,109	95.9	4,359	93.4	0.00
Non-white	301	4.1	308	6.6	
Missing	873	-	1,734	-	
**Parity**					
0	3,477	42.0	2,293	35.8	
1	2,720	32.8	1,819	28.4	0.00
≥2	1,362	16.4	1,253	16.4	
Missing	724	-	1,760	-	
**Maternal age**					
Under 25 years	1,687	21.6	2,602	42.4	0.00
Missing	455	15.6	257	4.0	
**Breastfeeding**					
Yes	3,346	40.4	1,787	27.9	
Missing	4,937	59.6	4,614	72.1	0.00
**Maternal smoking during pregnancy**					
Yes	1,371	20.0	1,529	36.0	0.00
Missing	1,429	-	2,157	-	
**Family Adversity Index at 2-4 years**					
<3	5,646	74.0	3,096	63.3	0.00
≥3	1,987	26.0	1,794	36.7	
Missing	651	-	1,511	-	
**Index of Multiple Deprivations at 7 years**					
1	2,079	33.0	318	23.2	0.00
≥2	4,213	67.0	1,054	76.8	
Missing	1,991	-	5,029	-	
**KS2 ≥ level 4**					
**English**					
Yes	6,172	75.0	3,616	56.9	0.00
Missing	995	-	1,653	-	
**Maths**					
Yes	5,962	82.6	3,536	69.1	0.00
Missing	1,050	-	1,212	-	
**Science**					
Yes	6,810	82.7	4,346	31.7	0.00
Missing	992	-	1,178	-	
**GCSEs ≥5 grade A*-C including Maths & English**					
Yes	4,177	59.5	1,875	37.2	0.00
**SEN at KS2**					
Yes (School action, Action plus or SEN)	948	13.7	1,162	22.8	
Missing	1,379	-	1,307	-	
**SEN at KS4**					
Yes (School action, Action plus or SEN)	733	11.3	859	18.2	
Missing	1,813	-	1,689	-	

Table ST2: Demographic characteristics of children that attended the ALSPAC research clinic at 7 years compared to those that did not attend. The student's T-test was used to calculate p-values.

Characteristic	Normal Vision & Hearing (n= 2,473)		Hearing Impairment alone (n=247)			Visual Impairment alone (n=175)			Co-occurring hearing & visual Impairment (n=14)		
	N	Percentage (%)	N	Percentage (%)	P-value	N	Percentage (%)	P-value	N	Percentage (%)	P-value
**Sex**<br>Male<br>Female	 1,218<br>1,255	 49.3<br>50.7	 125<br>122	 50.6<br>49.4	 0.68	 79<br>96	 45.1<br>54.9	 0.29	 10<br><5	 71.4<br>28.6	 0.10
**Low birthweight (<2,500g)**	89	3.6	10	4.1	0.72	11	6.3	0.07	<5	7.1	0.48
**Premature (gestation <37 weeks)**	121	4.9	16	6.5	0.28	11	6.3	0.41	<5	14.3	0.11
**Admission to SCBU**	134	5.4	23	9.3	0.01	11	6.3	0.62	<5	0	0.37
**Ethnicity**<br>White<br>Non-white	 2,398<br>75	 97.0<br>3.0	 241<br>6	 97.6<br>2.4	 0.60	 172<br><5	 98.3<br>1.7	 0.32	 14<br><5	 100<br>0	 0.51
**Parity**<br>0<br>1<br>≥2	 1,122<br>920<br>431	 45.4<br>37.2<br>17.4	 121<br>79<br>47	 49.0<br>32.0<br>19.0	 0.28<br>0.11<br>0.53	 77<br>78<br>20	 44.0<br>44.6<br>11.4	 0.73<br>0.05<br>0.04	 6<br>6<br><5	 42.9<br>42.9<br>14.3	 0.85<br>0.66<br>0.76
**Maternal age <25 years**	452	18.3	48	19.4	0.65	39	22.3	0.19	<5	14.3	0.70
**Breastfed**	1,103	44.6	106	42.9	0.61	85	48.6	0.31	<5	28.6	0.23
**Maternal smoking during pregnancy**	445	18.0	39	15.8	0.39	43	24.6	0.03	5	35.7	0.09
**Family Adversity Index at 2-4 years**<br><3<br>≥3	 2,089<br>384	 84.5<br>15.5	 205<br>42	 83.0<br>17.0	 0.54	 139<br>36	 79.4<br>20.6	 0.08	 11<br><5	 78.6<br>21.4	 0.54
**Index of Multiple Deprivations at 7 years**<br>1<br>≥2	 822<br>1,652	 33.2<br>66.8	 72<br>175	 29.2<br>70.9	 0.19	 54<br>121	 30.9<br>69.1	 0.52	 5<br>9	 35.7<br>64.2	 0.84

Table ST3: Demographic characteristics of children according to their hearing and vision status. NB. "<5" may include zero. The student's T-test was used to calculate p-values.

	KS2 English		KS2 Maths		KS2 Science	
	N	Percentage (95% CI)	N	Percentage (95% CI)	N	Percentage (95% CI)
**Normal vision & hearing N=2,473**	2,306	93.3<br>(92.3 – 94.2)	2,429	98.2<br>(97.7 – 98.7)	2,421	97.9<br>(97.3 – 98.5)
**Hearing impairment N= 247**	226	91.5<br>(88.0 – 95.0)	239	96.8<br>(94.5 – 99.0)	241	97.6<br>(95.6 – 99.5)
**Visual impairment N= 175**	149	85.1<br>(79.8 – 90.5	161	92.0<br>(87.9 – 96.1)	165	94.3<br>(90.8 – 97.8)
**Co-occurring hearing & visual impairment N=14**	10	71.4<br>(44.4 – 98.5)	12	85.7<br>(64.8 - >100)	13	92.9<br>(77.4 - >100)

Table ST4: Proportions of children achieving level 4 or above at Key Stage 2 English, Maths and Science by hearing and vision status

	Normal hearing & vision N = 2480	Hearing impairment N= 247	Visual impairment N= 175	Co-occurring hearing & visual impairment N=14	P-value
**SEN support at KS2**<br>Number<br>Percent (%)<br>(95% CI)	153<br>6.2<br>(5.2 – 7.7)	21<br>8.5<br>(5.4 – 12.3)	20<br>11.4<br>(7.0 – 16.3)	<5<br>14.3<br>(<0 – 35.3)	0.02
**SEN support at KS4**<br>Number<br>Percent (%)<br>(95% CI)	147<br>5.94<br>(5.0 – 6.9)	17<br>9.7%<br>(5.4 – 12.3)	21<br>11.05<br>(6.6 – 15.6)	<5<br>28.6<br>(1.5 – 55.6)	0.01

Table ST5: Proportions of children receiving SEN support at KS2 & KS4 by hearing and vision status. The Pearson's chi squared test was used to calculate p-values.

Table ST6: Multivariate regression models using the imputed dataset (N=8,205)

KS2 Maths: Achievement of ≥level 4												
	**Univariate logistic regression**		**Model 1** : Male, age at testing, SEN at KS2		**Model 2:** Model 1 & maternal age <25, parity & smoking during pregnancy		**Model 3:** Model 2 & FAI & IMD		**Model 4:** Model 3 & IQ		**Model 5:** Model 4 & behaviour, social perceptions & attention	
	**OR**	**95% CI**	**OR**	**95% CI**	**OR**	**95% CI**	**OR**	**95% CI**	**OR**	**95% CI**	**OR**	**95% CI**
**Hearing impairment** (n=852)	0.83	0.68 – 1.01	0.90	0.71 – 1.15	0.90	0.71 – 1.15	0.90	0.71 – 1.15	0.91	0.72 – 1.16	0.91	0.71 – 1.15
**Visual impairment** (n=573)	0.66	0.53 – 0.83	0.81	0.61 – 1.07	0.80	0.60 – 1.06	0.81	0.61 – 1.07	0.81	0.61 – 1.08	0.82	0.61 – 1.08
**Co-occurring hearing & visual impairment** (n=45)	0.33	0.17 – 0.63	0.47	0.22 – 1.01	0.46	0.21 – 1.01	0.46	0.21 – 1.01	0.46	0.21 – 1.00	0.46	0.21 – 1.00

demonstrating the likelihood children achieved ≥level 4 at KS2 Maths according to hearing and visual status and after adjustment for confounding variables (models 1-3) and mediating factors (models 4-5). OR=Odds Ratio

KS2 Science: Achievement of ≥level 4												
	**Univariate logistic regression**		**Model 1** : Male, age at testing, SEN at KS2		**Model 2:** Model 1 & maternal age <25, parity & smoking during pregnancy		**Model 3:** Model 2 & FAI & IMD		**Model 4:** Model 3 & IQ		**Model 5:** Model 4 & behaviour, social perceptions & attention	
	**OR**	**95% CI**	**OR**	**95% CI**	**OR**	**95% CI**	**OR**	**95% CI**	**OR**	**95% CI**	**OR**	**95% CI**
**Hearing impairment** (n=852)	0.89	0.73 – 1.08	0.97	0.77 – 1.22	0.97	0.77 – 1.22	0.97	0.77 – 1.22	0.99	0.78 – 1.24	0.99	0.78 – 1.24
**Visual impairment** (n=573)	0.69	0.54 – 0.87	0.83	0.64 – 1.08	0.84	0.65 – 1.09	0.84	0.65 – 1.10	0.86	0.66 – 1.12	0.87	0.66 – 1.13
**Co-occurring hearing & visual impairment** (n=45)	0.33	0.18 – 0.61	0.46	0.22 – 0.95	0.47	0.23 – 0.97	0.47	0.23 – 0.97	0.48	0.23 – 0.99	0.48	0.23 – 0.99

Table ST7: Multivariate regression models using the imputed dataset (N=8,205) demonstrating the likelihood children achieved ≥level 4 at KS2 Science according to hearing and visual status and after adjustment for confounding variables (models 1-3) and mediating factors (models 4-5). OR=Odds Ratio

KS2 English: Achievement of ≥level 4								
	**Univariate logistic regression**		**Model 1** : Male, age at testing, SEN at KS2		**Model 2:** Model 1 & maternal age <25, parity & smoking during pregnancy		**Model 3**: Model 2 & FAI & IMD	
	**OR**	**95% CI**	**OR**	**95% CI**	**OR**	**95% CI**	**OR**	**95% CI**
**Hearing impairment** (n=261)	0.74	0.48 – 1.15	0.81	0.50 – 1.32	0.80	0.49 – 1.31	0.76	0.47 – 1.24
**Visual impairment** (n=189)	0.39	0.30 – 0.60	0.42	0.26 – 0.67	0.42	0.26 – 0.68	0.42	0.26 – 0.68
**Co-occurring hearing & visual impairment** (n=14)	0.20	0.06 – 0.64	0.21	0.06 – 0.81	0.21	0.05 – 0.84	0.21	0.05 – 0.83
**KS2 Maths: Achievement of ≥level 4**								
	**Univariate logistic regression**		**Model 1** : Male, age at testing, SEN at KS2		**Model 2:** Model 1 & maternal age <25, parity & smoking during pregnancy		**Model 3:** Model 2 & FAI & IMD	
	**OR**	**95% CI**	**OR**	**95% CI**	**OR**	**95% CI**	**OR**	**95% CI**
**Hearing impairment** (n=261)	0.56	0.28 – 1.11	0.64	0.30 – 1.33	0.66	0.31 – 1.40	0.58	0.27 – 1.23
**Visual impairment** (n=189)	0.21	0.12 – 0.38	0.24	0.12 – 0.46	0.21	0.11 – 0.41	0.20	0.10 – 0.41
**Co-occurring hearing & visual impairment** (n=14)	0.14	0.03 – 0.64	0.15	0.02 – 0.91	0.13	0.02 – 0.87	0.10	0.02 – 0.57
**KS2 Science: Achievement of ≥level 4**								
	**Univariate logistic regression**		**Model 1** : Male, age at testing, SEN at KS2		**Model 2:** Model 1 & maternal age <25, parity & smoking during pregnancy		**Model 3:** Model 2 & FAI & IMD	
	**OR**	**95% CI**	**OR**	**95% CI**	**OR**	**95% CI**	**OR**	**95% CI**
**Hearing impairment** (n=261)	0.87	0.39 – 1.92	1.00	0.44 – 2.28	1.00	0.43 – 2.31	0.89	0.38 – 2.08
**Visual impairment** (n=189)	0.35	0.18 – 0.68	0.46	0.23 – 0.94	0.49	0.23 – 1.03	0.53	0.24 – 1.16
**Co-occurring hearing & visual impairment** (n=14)	0.31	0.04 – 2.42	0.38	0.04 – 3.57	0.49	0.05 – 5.05	0.35	0.04 – 3.21

Table ST8-ST10: Multivariate regression models using complete cases (N=2,909) demonstrating the likelihood children achieved ≥level 4 at KS2 English, Maths & Science according to hearing and visual status and after adjustment for confounding variables (models 1-3). OR=Odds Ratio

KS4: Achievement of ≥5 GCSEs at A*-C, including Maths & English										
	**Univariate logistic regression**		**Model 1 :** Male, SCBU admission, SEN at KS4		**Model 2:** Model 1 & maternal age <25, parity & smoking during pregnancy		**Model 3:** Model 2 & FAI & IMD		**Model 4:** Model 3 & KS2 English, Maths & Science performance	
	**OR**	**95% CI**	**OR**	**95% CI**	**OR**	**95% CI**	**OR**	**95% CI**	**OR**	**95% CI**
**Hearing impairment** (n=261)	0.66	0.51 – 0.86	0.69	0.52 – 0.91	0.66	0.50 – 0.88	0.64	0.48 – 0.86	0.64	0.47 – 0.87
**Visual impairment** (n=189)	0.91	0.66 – 1.26	1.00	0.71 – 1.41	1.08	0.76 – 1.53	1.12	0.77 – 1.59	1.47	0.98 – 2.21
**Co-occurring hearing & visual impairment** (n=14)	0.51	0.18 – 1.49	0.79	0.25 – 2.53	0.82	0.24 – 2.79	0.77	0.22 – 2.64	1.18	0.29 – 4.74

Table ST11: Multivariate regression models using complete cases (N=2,909) demonstrating the likelihood children achieved ≥level 4 at KS2 English, Maths & Science and ≥5 GCSEs at A-C grade (including Maths & English), according to hearing and visual status and after adjustment for. OR=Odds Ratio*
