# Supplementary material for: Impact of co-occurring hearing and visual difficulties in childhood on educational outcomes: a longitudinal cohort study
Source: BMJ Paediatr Open. 2019 Apr 3;3(1):e000389. doi: 10.1136/bmjpo-2018-000389 (PMC6542436; doi:10.1136/bmjpo-2018-000389)
Supplement: Author's manuscript [file bmjpo-2018-000389.draft_revisions.pdf]

BMJ Paediatrics Open

BMJ Paediatrics Open is committed to open peer review. As part of this commitment we make the peer review history of every article we publish publicly available.

When an article is published we post the peer reviewers' comments and the authors' responses online. We also post the versions of the paper that were used during peer review. These are the versions that the peer review comments apply to.

The versions of the paper that follow are the versions that were submitted during the peer review process. They are not the versions of record or the final published versions. They should not be cited or distributed as the published version of this manuscript.

BMJ Paediatrics Open is an open access journal and the full, final, typeset and author-corrected version of record of the manuscript is available on our site with no access controls, subscription charges or pay-per-view fees (http://bmjpaedsopen.bmj.com).

If you have any questions on BMJ Paediatrics Open's open peer review process please email info.bmjpo@bmj.com

BMJ Paediatrics Open

THE IMPACT OF CO-OCCURING HEARING AND VISUAL IMPAIRMENTS IN CHILDHOOD ON EDUCATIONAL OUTCOMES: A LONGITUDINAL COHORT STUDY

Journal:	*BMJ Paediatrics Open*
Manuscript ID	bmjpo-2018-000389
Article Type:	Original article
Date Submitted by the Author:	16-Oct-2018

Complete List of Authors:	Hill, Matilda; University of Bristol Centre for Child and Adolescent Health<br>Hall, Amanda; Aston University School of Life and Health Sciences<br>Williams, Cathy; University of Bristol Centre for Child and Adolescent Health<br>Emond, Alan; University of Bristol, Centre for Child and Adolescent Health

Keywords:	Comm Child Health, Ophthalmology, Audiology, Epidemiology, Paediatric Practice

SCHOLARONE™
Manuscripts

THE IMPACT OF CO-OCCURING HEARING AND VISUAL IMPAIRMENTS IN CHILDHOOD ON EDUCATIONAL OUTCOMES: A LONGITUDINAL COHORT STUDY

Matilda Hill[1], Amanda Hall[2], Cathy Williams[1], Alan Emond[1]

matildahill84@gmail.com, amanda.hall@bristol.ac.uk, cathy.williams@bristol.ac.uk, alan.emond@bristol.ac.uk

Correspondence to: Dr Matilda Hill, Centre for Academic Child Health, Bristol Medical School, University of Bristol BS8 1NU

Word Count: 3,405

[1] Centre for Academic Child Health, Bristol Medical School, University of Bristol BS8 1NU
[2] Life and Health Sciences, Aston University, Birmingham B4 7ET

Confidential: For Review Only

Article Summary

What is known about the subject

- Mild hearing and visual impairments are common in childhood.
- The most common cause of hearing impairments in childhood is chronic otitis media with effusion (OME), which may be associated with lower academic performance.
- The impact of common co-occurring hearing and visual impairments on educational outcomes is not known.

What this study adds

- Children with co-occurring mild hearing and visual impairments have poorer educational outcomes than children with isolated hearing or visual impairments.
- Mild visual impairments alone do not negatively impact on academic performance at school.

Confidential: For Review Only

Abstract

Background: Mild hearing and visual impairments are common in childhood, and both may have implications for educational achievement. However, the impact of co-occurring common hearing and visual impairments in childhood is not known.

Objective: To determine the prevalence and impact of co-occurring common hearing and visual impairments of childhood on educational outcomes in primary and secondary school.

Methods: The sample was drawn from the Avon Longitudinal Study of Parents and Children (ALSPAC), a longitudinal birth cohort study in England. The exposures were hearing and visual impairments at age 7 (defined as conductive hearing loss or OME, and amblyopia, strabismus or reduced visual acuity, respectively). The outcomes measured were achievement of level 4 or above at Key Stage 2 (KS2) in English, Maths and Science at age 11, and attainment of 5 or more GCSEs at grades A*-C at age 16. Multivariate regression models assessed the relationship between hearing and visual impairments and educational outcomes, adjusting for potential confounding factors.

Results: Children with co-occurring hearing and visual difficulties were less likely to achieve the national target at KS2 compared to children with normal hearing and vision, even after adjustment for confounding factors. Differences in IQ, behaviour, attention and social cognition did not account for this relationship. The impact of co-occurring hearing and visual impairments on GCSE results was largely explained by poor performance at KS2.

Conclusions: Co-occurring hearing and visual impairments in childhood have an enduring negative impact on educational outcomes. Identification of affected children and early intervention in primary school is essential.

Confidential: For Review Only

Confidential: For Review Only

Introduction

Hearing and visual difficulties are prevalent amongst children in the UK. The majority of affected children have mild, temporary impairments, which may not be formally diagnosed and for which they do not receive additional educational support. However, there is evidence that even mild deficits in hearing or vision can have implications for learning and development throughout childhood(1-5).

The most common cause of chronic hearing loss in childhood is persistent otitis media with effusion (OME, 'glue ear'). There is ongoing controversy regarding the impact of OME on language development and cognition. Whilst several large studies have found no association between OME and language or academic attainment(6, 7), few of these studies have considered concurrent hearing loss or chronicity of OME. Conversely, a number of studies have demonstrated an association between persistent OME-related hearing loss, cognitive development, reading ability and behavioural problems (2-4, 8).

The impact of common visual impairments on academic attainment in childhood is not well characterised(9). Decreased visual acuity in childhood is associated with reduced literacy(1), and it may be hypothesised that other impairments to vision may also affect reading ability.

The impact of co-occurring common hearing and visual impairments on educational outcomes in childhood has not previously been studied. However, it is well-established that integration of auditory and visual information is essential for many cognitive processes, in particular speech perception, and key for the development of language and communication skills(10, 11). A deficit in one of these domains in childhood may therefore hinder developing literacy and render an individual vulnerable to the cumulative effect of an additional sensory deficit.

Confidential: For Review Only

The aim of this study is to investigate the prevalence and impact of co-occurring common hearing and visual impairments in childhood on educational outcomes at primary school and secondary school using a large population cohort. We hypothesise that children with co-occurring mild hearing and visual impairments are more likely to have poorer language and communication skills, and thus lower levels of academic attainment, relative to those with a single sensory deficit or children with normal hearing and vision.

Methods

Study Participants

The Avon Longitudinal Study of Parents and Children (ALSPAC) is a longitudinal population-based birth cohort study. All pregnant women resident in Avon, UK, with expected dates of delivery between 1st April 1991 and 31st December 1992 were eligible for participation, resulting in enrolment of over 14,000 live births(12, 13). Ethical approval for the study was obtained from the ALSPAC Ethics and Law Committee and the Local Research Ethics Committees.

Please note that the study website contains details of all the data that is available through a fully searchable data dictionary and variable search tool: http://www.bristol.ac.uk/alspac/researchers/our-data/

Patient and Public Involvement

ALSPAC participants advise on ALSPAC studies through the original cohort advisory panel (OCAP) and contribute to the ALSPAC ethics and law committee. ALSPAC participants were not directly involved in the design of this study.

Hearing and vision assessments

All participating children in ALSPAC were invited to attend a research clinic at 7 years of age. 8,299 children attended during the period September 1998 - September 2000, of whom 8,205 were eligible for

Confidential: For Review Only

inclusion (see Figure 1). The clinics included a comprehensive assessment of vision and hearing, details of which are included in Appendix A of the Supplementary Material.

Definition of Hearing Impairment

Hearing impairment was defined as the presence of conductive hearing loss and/or OME in either ear, characterised by air conduction greater than 20 dB averaged across 500 Hz, 1kHz, 2kHz and 4kHz, or the presence of a type B tympanogram, respectively. The prevalence of OME decreases with age after the first 2 years of life(14); children with evidence of OME at age 7 are likely to have persistent OME which is associated with conductive hearing loss in over 70% of cases(15, 16). However, hearing loss may be fluctuant and thus not captured by a single clinic assessment, hence the utilisation of both air conduction tests and tympanometry to identify children with hearing impairments.

A total of 5 children with sensorineural hearing loss, defined by bone conduction greater than 30 dB at either 1 kHz or 4 kHz, were excluded from this analysis.

Definition of Visual Impairments

Children with "clinically significant" strabismus, amblyopia or reduced acuity were defined as having visual difficulties. "Clinically significant" strabismus comprised all children with manifest strabismus, or previously defined large latent deviations (>=10 pd if convergent & >=15pd if divergent)(17). Amblyopia was defined as a history of patching treatment and/or an interocular difference in acuity of >0.2logMAR units where the worst-seeing eye had an acuity of >0.3logMAR. Reduced acuity was defined by reduced distance acuity of the better-seeing eye >=0.3 logMAR.

Refractive errors were not included in our definition of visual difficulties as these are potentially correctable with glasses and have already been studied extensively in the context of educational achievement, with myopia being linked with higher educational achievement(18, 19).

Confidential: For Review Only

Triplets, quadruplets and children with known ophthalmic pathology, Down's Syndrome or Cerebral Palsy were excluded from this analysis.

Educational Outcomes

Educational outcomes at primary school were assessed using Standardised Assessment Test (SAT) results, obtained from the National Pupil Database (NPD)(20). Key Stage 2 (KS2) tests are undertaken during the final year of primary school (year 6) at age 10-11, with pupils being expected to achieve National Curriculum level 4 or above. We used achievement of ≥level 4 at KS2 English, Maths and Science as our educational outcomes in primary school.

Educational outcomes in secondary school were assessed using General Certificate of Secondary Education (GCSE) results at Key Stage 4 (KS4). GCSEs are taken at the end of compulsory schooling (year 11) at age 16, and are graded A*- G. Achievement of 5 or more GCSEs (including Maths and English) at grades A*- C is widely used as a marker of educational performance, and we used this as our outcome at secondary school. We adjusted outcomes for KS2 attainment, as performance at KS2 is well known to predict performance at GCSEs(21).

Information on children receiving Special Educational Needs (SEN) support was provided by the Pupil Level Annual School Census (PLASC). SEN status was obtained for each child at KS2 and KS4, with information on the level of individual educational support being provided. We dichotomised these data into children receiving no special provision and those receiving some level of support.

NPD and PLASC data were not available for children attending independent schools, or schools outside of England, and these children were excluded from this analysis.

Confidential: For Review Only

Confounding and Mediating Factors

Gender, prematurity, low birthweight, admission to a Special Care Baby Unit (SCBU), maternal age, parity, smoking during pregnancy, duration of breastfeeding and socioeconomic status were selected as potential confounding factors, based on their previously identified associations with hearing or visual impairments in childhood and well-established links with academic performance(17, 22-27).

The ALSPAC Family Adversity Index (FAI), derived from a questionnaire about socioeconomic status completed by mothers at 2-4 years, was used as a measure of family adversity. The Indices of Multiple Deprivations (IMD) at age 7, a census-derived score of relative deprivation of a neighbourhood was used as a measure of social deprivation.

Intelligence quotient (IQ), attention, social cognition and behaviour were selected as potential mediating factors, as they may be influenced by visual and hearing impairments and can impact on educational achievement(1, 3, 4, 8). Reading ability was not included due to high rates of missing data. These domains were tested during a research clinic to which all participating children were invited to attend at 8 years of age. Further information on these assessments is provided in Appendix B of the Supplementary Material.

Analyses

The Pearson's chi-squared test, the Student's t-test and the Mann-Whitney U test were used to assess for differences between groups. Binary univariate logistic regressions were used to calculate odds ratios (OR) and 95% confidence intervals (95% CI) to assess the relationship between hearing and visual impairments and educational outcomes. The analyses were repeated controlling for all potential confounding factors and mediating factors significant at the 5% level in the univariate analyses, using a series of multivariate logistic regression models. These sequentially adjusted for individual factors, maternal factors, wider socioeconomic factors, earlier educational performance and potential mediating factors. Further information is provided in Tables 2 & 3.

Confidential: For Review Only

Multiple imputation using chained equations (ICE) was used to impute missing data for all variables included in the final logistic regression models, including the outcome, and variables that predicted missingness. This technique helps to minimise attrition bias and improve precision of estimates(28). We imputed data for all 8,205 children who attended the research clinic at 7 years and did not meet the exclusion criteria. Twenty imputations were performed. All analyses were carried out using STATA version 15.0 (StataCorp 2017). Further information regarding the multiple imputation is provided in Appendix C of the Supplementary Material.

Results

Sample Characteristics

Of the 8,205 children that attended the research clinic at 7 years and were eligible for inclusion, 2,909 (33.5%) had complete data for hearing and vision, educational outcomes and potential confounding variables (see Figure 1).

Children attending the research clinic were more likely to be come from families of higher socioeconomic class and achieve higher levels of academic attainment at both KS2 and KS4 than children that did not attend the clinic (see supplementary material, Table ST2).

Prevalence and Causes of Hearing & Visual Impairments

Of the 2,909 children with complete data, 261 (9.0%) had hearing impairments, 189 (6.5%) had visual difficulties and 14 (0.5%) had both hearing and visual impairments.

Confidential: For Review Only

Types of hearing and visual impairment are demonstrated in Table 1. The majority of children with conductive hearing loss had evidence of concurrent OME. The overall prevalence of OME in either or both ears was 6.7%. A higher proportion of children undergoing assessment in winter months had OME compared to those attending clinic in the summer months (8.0% vs 5.6%), but this association was weak (p=0.09).

Types of Hearing and Visual Impairments	Proportion of children with either hearing or visual impairments (%)
**Hearing Impairments**	**261 (100%)**
Otitis Media with Effusion	102 (39.1%)
Otitis Media with Effusion & Conductive Hearing Loss	94 (36.0%)
Conductive Hearing Loss	65 (24.9%)
**Visual Impairments**	**189 (100%)**
Amblyopia	79 (41.8%)
Strabismus	56 (29.6%)
Strabismus & Amblyopia	47 (24.9%)
Reduced Acuity with or without Amblyopia and/or Strabismus	7 (3.7%)

Confidential: For Review Only

Table 1: Types of hearing and visual impairment

Amblyopia was the most common cause of visual impairment, affecting 4.4% of all children There was considerable overlap between amblyopia and strabismus; of the 128 children with amblyopia, 47 (36.7%) also had strabismus. Very few children had reduced acuity in the best-seeing eye.

Children with hearing and visual impairments had similar characteristics to those with normal hearing and vision (see supplementary material, Table ST3). However, children with visual difficulties were more likely to have mothers who smoked during pregnancy (24.6% vs 18.0%, p=0.03) and children with hearing impairments were more likely to have been admitted to SCBU in infancy (9.3% vs 5.4%, p=0.01).

Educational Outcomes and SEN status in Primary School

Overall, 92.5% and 97.7% of children achieved level 4 or above in KS2 English and Maths tests, respectively. The proportions of children achieving these targets by hearing and visual status are shown in supplementary material, Table ST4.

Children with either hearing or visual impairments were less likely to achieve level 4 or above at KS2 English tests relative to children with normal hearing and vision (see Table 2 for the imputed dataset analysis; see supplementary material, Table ST8-ST10 for the complete cases analysis). However, these relationships were attenuated by adjustment for confounding factors, and further attenuated after adjustment for IQ, suggesting that some of the association is mediated through IQ. In contrast, children with co-occurring hearing and visual impairments remained less likely to achieve this target after controlling for confounding factors (OR 0.30, CI 0.15-0.61). This relationship was not significantly altered by adjustment for IQ or other potential mediating factors.

Confidential: For Review Only

Confidential: For Review Only

KS2 English: Achievement of ≥level 4												
	**Univariate logistic regression**		**Model 1** : Male, age at testing, SEN at KS2		**Model 2:** Model 1 & maternal age <25, parity & smoking during pregnancy		**Model 3:** Model 2 & FAI & IMD		**Model 4:** Model 3 & IQ		**Model 5:** Model 4 & behaviour, social perceptions & attention	
	**OR**	**95% CI**	**OR**	**95% CI**	**OR**	**95% CI**	**OR**	**95% CI**	**OR**	**95% CI**	**OR**	**95% CI**
**Hearing impairment** (n=852)	0.78	0.66 – 0.92	0.80	0.66 – 0.98	0.81	0.66 – 0.99	0.81	0.66 – 0.99	0.87	0.71 – 1.06	0.88	0.72 – 1.08
**Visual impairment** (n=573)	0.66	0.64 – 0.80	0.79	0.62 – 0.99	0.81	0.64 – 1.02	0.82	0.64 – 1.03	0.87	0.68 – 1.11	0.90	0.71 – 1.14
**Co-occurring hearing & visual impairment** (n=45)	0.25	0.14 – 0.45	0.30	0.15 – 0.61	0.30	0.15 – 0.61	0.30	0.15 – 0.61	0.36	0.17 – 0.74	0.38	0.18 – 0.78

Table 2: Multivariate regression models using the imputed dataset (N=8,205) demonstrating the likelihood children achieved ≥level 4 at KS2 English according to hearing and visual status and after adjustment for confounding variables (models 1-3) and mediating factors (models 4-5). OR=Odds Ratio

The relationship is similar for performance in KS2 Maths and KS2 Science tests, albeit hearing impairments appear to have less of an impact (see supplementary material, Table ST6 and ST7).

The proportion of children with visual impairments receiving SEN support was higher compared to children with normal hearing and vision (11.4% vs 6.2%, p =0.02, see supplementary material, Table ST5). Overall, children with SEN status at KS2 were less likely to achieve level 4 or above in KS2 English, Maths and Science tests than those without formal support (52.6% vs 87.6%, p=0.00).

Educational Outcomes and SEN Status in Secondary School

Overall, almost three-quarters (72.1%) of the study sample achieved 5 or more GCSEs (including English and Maths) at A*-C grade. The proportion of children with hearing impairments achieving this target was lower compared to those with normal hearing and vision (see Figure 2).

**KS4: Achievement of ≥5 GCSEs at A*-C, including Maths & English**														
	**Univariate logistic regression**		**Model 1 :** Male, SCBU admission, SEN at KS4		**Model 2:** Model 1 & maternal age <25, parity & smoking during pregnancy		**Model 3:** Model 2 & FAI & IMD		**Model 4:** Model 3 & KS2 English, Maths & Science performance		**Model 5:** Model 4 & IQ		**Model 6**: Model 5 & behaviour, social perceptions & attention	
	**OR**	**95% CI**	**OR**	**95% CI**	**OR**	**95% CI**	**OR**	**95% CI**	**OR**	**95% CI**	**OR**	**95% CI**	**OR**	**95% CI**
**Hearing impairments** (n=852)	0.80	0.68 – 0.94	0.83	0.69 – 0.99	0.83	0.69 – 0.99	0.82	0.68 – 0.99	0.85	0.69 – 1.04	0.95	0.76 – 1.17	0.96	0.77 – 1.19

Confidential: For Review Only

**Visual impairments** (n=573)	0.86	0.71 – 1.05	0.94	0.74 – 1.17	1.00	0.79 – 1.27	1.02	0.81 – 1.30	1.20	0.92 – 1.56	1.34	1.00 – 1.79	1.37	1.02 – 1.84
**Co-occurring hearing & visual impairments** (n=45)	0.55	0.29 – 1.06	0.67	0.29 – 1.51	0.68	0.29 – 1.58	0.66	0.28 – 1.53	1.13	0.43 – 2.94	1.42	0.50 – 4.06	1.49	0.52 – 4.29

Table 3: Multivariate regression models using the imputed dataset (N=8,205) demonstrating the likelihood children achieved ≥5 GCSEs at A-C grade (including Maths & English), according to hearing and visual status and after adjustment for confounding variables (models 1-3) and mediating factors (models 4-5). OR=Odds Ratio*

The association between hearing impairments and poorer performance at GCSEs is attenuated after adjustment for performance at KS2 and IQ, suggesting that the association between hearing and education is mediated through these factors (see Table 3 for the imputed dataset analysis; see supplementary material, Table ST11 for the complete cases analysis). Children with visual difficulties alone were no less likely to attain 5 or more GCSEs at A*-C. Children with co-occurring hearing and visual impairments were less likely to achieve this target even after adjustment for confounding factors, although this association was weak(OR 0.66, CI 0.28-1.53). This relationship is attenuated after adjustment for performance at KS2, suggesting that poorer outcomes at KS4 in these children can be partly explained by poorer educational outcomes at primary school.

At KS4, the overall proportion of children receiving SEN support was similar to that at KS2 (6.7% vs 6.5%, p=0.76). Children with visual impairments were almost twice as likely to receive SEN support than those with normal hearing and vision (see supplementary material, Table ST5). Children with SEN provision were less likely to attain 5 GCSEs at A*-C (32.3% vs 74.9%, p=0.00).

Discussion

Confidential: For Review Only

Discussion of results

In this longitudinal study we have demonstrated that co-occurring mild hearing and visual impairments in childhood have a negative impact on educational outcomes, greater than the effect of hearing or visual impairments alone. We have shown a weak association between mild hearing impairments at age 7 and academic achievement at KS2 and KS4, which may be mediated by IQ. We found no association between mild visual impairments and academic outcomes. In contrast, children with co-occurring hearing and visual impairments were less likely to attain the expected academic standards at the end of primary school, an effect which is not wholly mediated through in IQ, behaviour, attention or social cognition, suggesting a substantial, unexplained educational disadvantage. They were also less likely to achieve 5 or more GCSEs at Key Stage 4, which may be attributable to poor performance at KS2. However, this relationship is weak, likely due to the relatively low prevalence of co-occurring mild sensory impairments and potential small effect size.

A higher proportion of children with visual impairments had SEN status at both primary school and secondary school relative to those with normal hearing and vision. Common visual impairments, as defined by this study, are of themselves not sufficient to merit SEN support, suggesting a higher prevalence of additional disabilities amongst children with visual impairments. This is consistent with previously reported findings(29). Children receiving SEN support are less likely to achieve the national targets at both primary school and secondary school compared to those without SEN support, implying SEN status is a proxy indicator of severity of educational impairment.

Strengths and Weaknesses

The major strength of this study is that it uses a large population-based birth cohort, with an accurate assessment of hearing and vision. Furthermore, it uses standardised national tests as objective outcome measures, and longitudinally collected data on a wide range of confounders.

Confidential: For Review Only

The most important limitation of this study relates to missing data and the under-representation of children from ethnic minorities and those from lower socio-economic backgrounds. A multiple imputation technique was utilised to minimise potential bias and improve precision. An additional limitation is the single time-point used to assess hearing, and possible inclusion of children with transient OME as a result, which may have led to underestimation of the effect of persistent OME-related hearing loss on academic attainment. Furthermore, we did not have information regarding which children were treated for their hearing and visual impairments either prior or following the assessment at age 7. Finally, whilst we excluded children with known diagnoses of Down's Syndrome or Cerebral Palsy, we did not have a full data regarding developmental disorders or medical co-morbidities. However, such conditions affect only a small minority of children with hearing or visual impairments and this was a population-based study designed to evaluate the impact of common, mild impairments(27, 30).

In the context of other research

Previous studies investigating the impact of co-occurring hearing and visual impairments in childhood have focused on children with severe deficits. Developmental disorders commonly co-occur with severe hearing and visual impairments, or 'deaf-blindness'(29-31), which often precludes children from attending mainstream schools and has additional implications for their education. To our knowledge, our study is the first to investigate the impact of co-occurring common mild hearing and visual impairments upon academic attainment, in a population of children attending mainstream schools.

The impact of childhood OME on cognition and educational performance has been studied previously by prospective cohort studies, although there is no clear consensus. Data from the longitudinal Dunedin study demonstrated an association between childhood OME and lower IQ in early teenage years(2), which is consistent with findings from the Aarhus Birth Cohort that reported hearing loss at 9 -11 years was associated with behavioural problems and reading difficulties(8). In contrast, data from the Danish

Confidential: For Review Only

National Birth Cohort showed no association between childhood OME episodes and school performance, findings supported by a meta-analysis of prospective studies, which found little to no association between OME and speech and language development(6, 7). The disparity in these findings is likely explained by the heterogeneity in study design, including inconsistencies in adjustment for confounding variables and consideration of associated hearing loss and persistence of OME. Our findings demonstrated a small effect of OME and/or hearing loss at age 7 on academic performance at age 10-11 and 16 years after adjustment for confounding variables, mediated through IQ. This is consistent with data from the 1970 British Birth Cohort(3), and previously published work using ALSPAC data, which have shown an association between OME and lower IQ(4), although their results suggest this association diminishes with age.

Previous research investigating the impact of common visual impairments in childhood on educational outcomes has focused largely on refractive errors, which were not included in this study(18, 19). Nonetheless, there is convincing evidence from a cross-sectional study involving participants of the Born in Bradford birth cohort study that reduced visual acuity at age 4-5 years is associated with reduced literacy(1). However, the prevalence of reduced visual acuity was considerably lower in our study (<1% vs 4%, likely due to the different socioeconomic and ethnic demographics of the cohorts). Data from the 1958 British Birth Cohort demonstrated no impact of unilateral amblyopia in childhood on educational tests at age 7, 11 and 16, or on highest educational qualification obtained(32). Amblyopia was the most common cause of visual impairment in our study, and our findings are consistent with this. Finally, we demonstrated higher rates of SEN amongst children with visual impairments; this is consistent with previous research demonstrating that visual impairments commonly co-occur with developmental disorders(30).

Clinical and Research Implications

Confidential: For Review Only

This study has important clinical implications. Children with known visual or hearing impairments should be routinely tested for additional sensory impairments, as even mild co-occurring visual or hearing deficits are associated with poorer educational outcomes. The impact of co-occurring hearing and visual impairments on performance at secondary school is largely explained by poor performance at primary school, hence early identification and intervention is essential.

Future research involving larger numbers of participants are required to replicate these findings.

Acknowledgements

We are extremely grateful to all the families who took part in this study, the midwives for their help in recruiting them, and the whole ALSPAC team, which includes interviewers, computer and laboratory technicians, clerical workers, research scientists, volunteers, managers, receptionists and nurses. The authors thank Dr Linda Hollen for providing advice on the statistical methodology.

Funding Statement

The UK Medical Research Council, the Wellcome Trust (Grant ref: 102215/2/13/2) and the University of Bristol provide core support for ALSPAC. A comprehensive list of grants funding is available on the ALSPAC website: http://www.bristol.ac.uk/alspac/external/documents/grant-acknowledgements.pdf

Author Contributors

MH, AH, CW & AE designed the study. MH carried out the data analysis & wrote the initial manuscript draft. AH, CW & AE contributed to the analysis, and reviewed and revised the manuscript. The authors declare that there is no conflict of interest in the publication of this research.

This publication is the work of the authors, who will serve as guarantors for the contents of this paper.

Confidential: For Review Only

References

1. Bruce A, Fairley L, Chambers B, Wright J, Sheldon TA. Impact of visual acuity on developing literacy at age 4-5 years: a cohort-nested cross-sectional study. BMJ Open. 2016;6(2):e010434.

2. Bennett KE, Haggard MP, Silva PA, Stewart IA. Behaviour and developmental effects of otitis media with effusion into the teens. Archives of disease in childhood. 2001;85(2):91-5.

3. Bennett KE, Haggard MP. Behaviour and cognitive outcomes from middle ear disease. Archives of disease in childhood. 1999;80(1):28-35.

4. Hall AJ, Maw R, Midgley E, Golding J, Steer C. Glue ear, hearing loss and IQ: an association moderated by the child's home environment. PloS one. 2014;9(2):e87021.

5. Archbold S YZ, Harrigan S Gregory S, Wakefield t, Holland L, Mulla I. Experiences of young people with mild to moderate hearing loss. National Deaf Children's Society; 2015.

6. Fougner V, Korvel-Hanquist A, Koch A, Dammeyer J, Niclasen J, Lous J, et al. Early childhood otitis media and later school performance - A prospective cohort study of associations. Int J Pediatr Otorhinolaryngol. 2017;94:87-94.

7. Roberts JE, Rosenfeld RM, Zeisel SA. Otitis media and speech and language: a meta-analysis of prospective studies. Pediatrics. 2004;113(3 Pt 1):e238-48.

8. Niclasen J, Obel C, Guldager C, Pleinert S, Dammeyer J. Associations between childhood hearing loss and behavioural and academic difficulties: A Danish cohort study. Int J Pediatr Otorhinolaryngol. 2016;90:91-8.

Confidential: For Review Only

9. Carlton J, Karnon J, Czoski-Murray C, Smith KJ, Marr J. The clinical effectiveness and cost-effectiveness of screening programmes for amblyopia and strabismus in children up to the age of 4-5 years: a systematic review and economic evaluation. Health technology assessment (Winchester, England). 2008;12(25):iii, xi-194.

10. Erdener D, Burnham D. The relationship between auditory-visual speech perception and language-specific speech perception at the onset of reading instruction in English-speaking children. Journal of experimental child psychology. 2013;116(2):120-38.

11. McGurk H, MacDonald J. Hearing lips and seeing voices. Nature. 1976;264(5588):746-8.

12. Boyd A, Golding J, Macleod J, Lawlor DA, Fraser A, Henderson J, et al. Cohort Profile: the 'children of the 90s'--the index offspring of the Avon Longitudinal Study of Parents and Children. Int J Epidemiol. 2013;42(1):111-27.

13. Fraser A M-WC, Tilling K, Boyd A, Golding J, Davey Smith G, Henderson J, Macleod J,, Molloy L NA, Ring S, Nelson SM, Lawlor DA. Cohort Profile: The Avon Longitudinal Study of Parents and Children: ALSPAC mothers cohort. International Journal of Epidemiology. 2013;42:97-110.

14. Midgley EJ, Dewey C, Pryce K, Maw AR. The frequency of otitis media with effusion in British pre-school children: a guide for treatment. ALSPAC Study Team. Clinical otolaryngology and allied sciences. 2000;25(6):485-91.

15. Daly KA, Hunter LL, Giebink GS. Chronic otitis media with effusion. Pediatrics in review. 1999;20(3):85-93.

16. Bluestone CD. Otitis Media in Infants and Children. 4th ed: People's Medical Publishing House--USA LTD; 2006.

Confidential: For Review Only

17. Williams C, Northstone K, Howard M, Harvey I, Harrad RA, Sparrow JM. Prevalence and risk factors for common vision problems in children: data from the ALSPAC study. Br J Ophthalmol. 2008;92(7):959-64.

18. Saw SM, Tan SB, Fung D, Chia KS, Koh D, Tan DT, et al. IQ and the association with myopia in children. Invest Ophthalmol Vis Sci. 2004;45(9):2943-8.

19. Mutti DO, Mitchell GL, Moeschberger ML, Jones LA, Zadnik K. Parental myopia, near work, school achievement, and children's refractive error. Invest Ophthalmol Vis Sci. 2002;43(12):3633-40.

20. Education TDf. National Pupil Database, UK data archive: Colchester, Essex.

21. Benton T ST. Analysis of use of Key Stage 2 data in GCSE predictions. ARD Research Division; 2014.

22. Paradise JL, Rockette HE, Colborn DK, Bernard BS, Smith CG, Kurs-Lasky M, et al. Otitis media in 2253 Pittsburgh-area infants: prevalence and risk factors during the first two years of life. Pediatrics. 1997;99(3):318-33.

23. Robaei D, Kifley A, Gole GA, Mitchell P. The impact of modest prematurity on visual function at age 6 years: findings from a population-based study. Archives of ophthalmology (Chicago, Ill : 1960). 2006;124(6):871-7.

24. Cumberland PM, Pathai S, Rahi JS, Millennium Cohort Study Child Health G. Prevalence of eye disease in early childhood and associated factors: findings from the millennium cohort study. Ophthalmology. 2010;117(11):2184-90 e1-3.

25. Sirin SR. Socioeconomic Status and Academic Achievement: A Meta-Analytic Review of Research. Review of Educational Research. 2005;75(3):417-53.

Confidential: For Review Only

26. Bowatte G, Tham R, Allen KJ, Tan DJ, Lau M, Dai X, et al. Breastfeeding and childhood acute otitis media: a systematic review and meta-analysis. Acta Paediatr. 2015;104(467):85-95.

27. Pathai S, Cumberland PM, Rahi JS. Prevalence of and early-life influences on childhood strabismus: findings from the Millennium Cohort Study. Archives of pediatrics & adolescent medicine. 2010;164(3):250-7.

28. Sterne JA, White IR, Carlin JB, Spratt M, Royston P, Kenward MG, et al. Multiple imputation for missing data in epidemiological and clinical research: potential and pitfalls. Bmj. 2009;338:b2393.

29. Salt A, Sargent J. Common visual problems in children with disability. Archives of disease in childhood. 2014;99(12):1163-8.

30. Kancherla V, Van Naarden Braun K, Yeargin-Allsopp M. Childhood vision impairment, hearing loss and co-occurring autism spectrum disorder. Disabil Health J. 2013;6(4):333-42.

31. NDCS. Prevalence of additional disabilities with deafness: A review of the literature. 2012.

32. Rahi JS, Cumberland PM, Peckham CS. Does amblyopia affect educational, health, and social outcomes? Findings from 1958 British birth cohort. BMJ. 2006;332(7545):820-5.

Confidential: For Review Only

Figure Legends

Figure 1: Study sample flow chart showing inclusion & exclusion criteria

Figure 2: Graph showing proportion of students achieving ≥5 GCSEs at A-C (including Maths & English) by hearing and vision status*

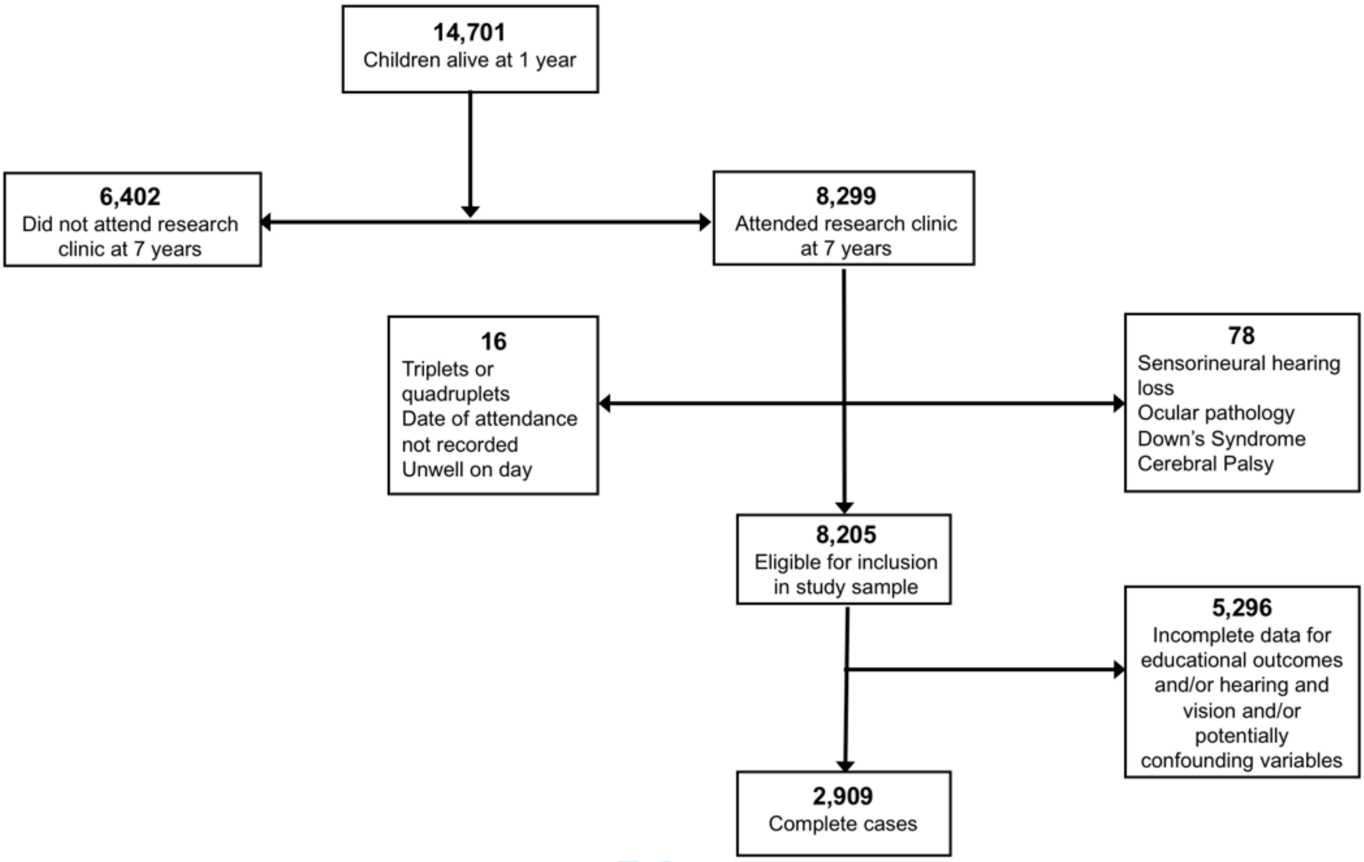

For Review Only

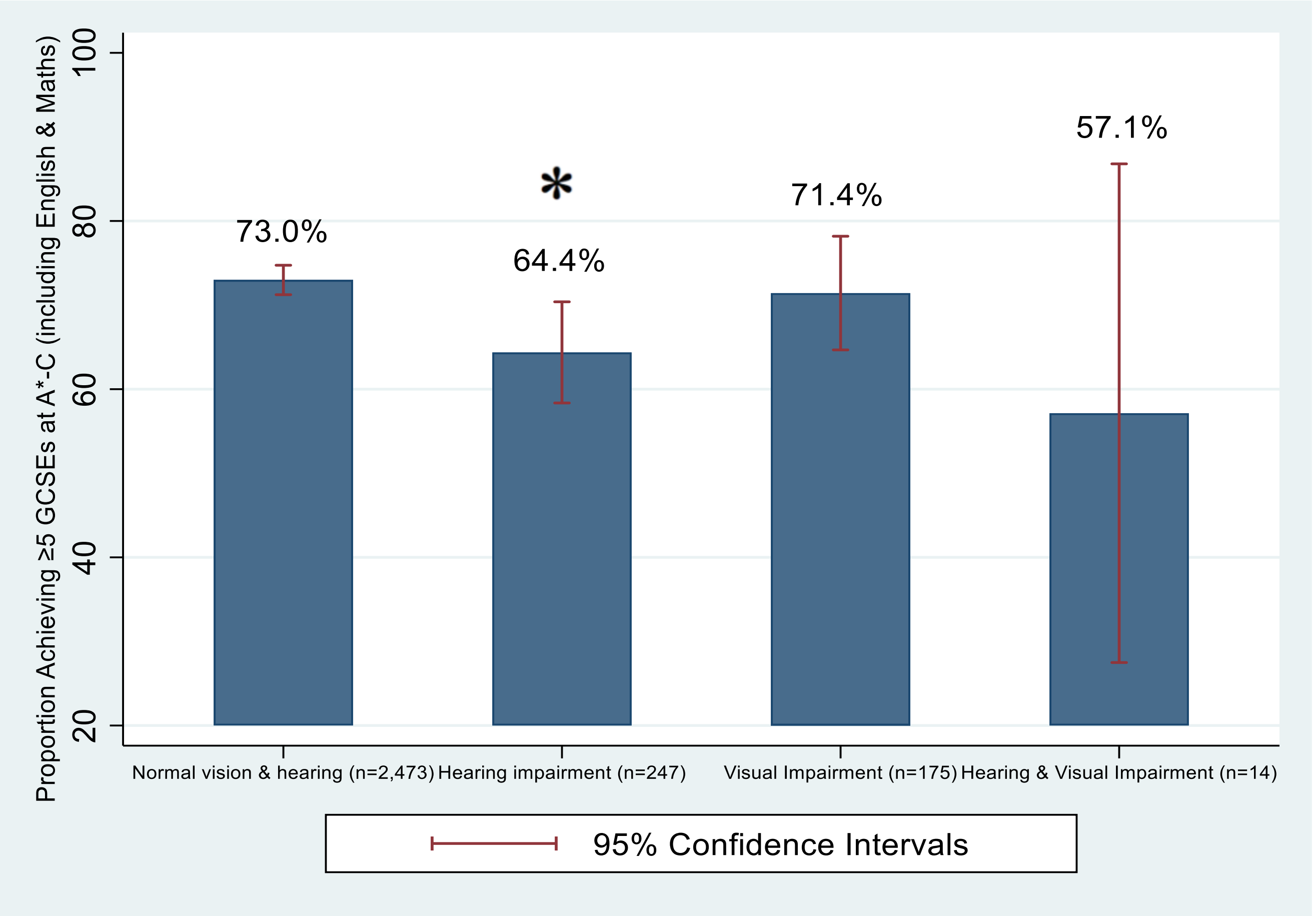

For Review Only

Confidential: For Review Only

Supplementary Material

Appendix A Hearing and vision assessment

The hearing assessment was carried out by audiologists and specially trained staff, and performed as per the British Society of Audiologists standards(13). Air conduction hearing thresholds were measured bilaterally at 500 Hz, 1kHz, 2kHz, 4kHz and 8kHz, and bone conduction thresholds were measured at 4 kHz for all children and at 1kHz if time permitted. Tympanometry was used to measure middle ear function bilaterally. Tympanograms were categorised according to the Jerger's modified Fieullau-Nikolajsen method(14), with type B tympanograms indicating the presence of otitis media with effusion (OME, 'glue ear').

The visual tests were undertaken by trained orthoptists and overseen by an ophthalmologist. Visual acuity was measured monocularly using the LogMAR test and was taken as the best of presenting (with glasses if worn) and presenting-plus-pinhole. Ocular misalignment was assessed using a simultaneous prism cover test and alternate prism cover test, and strabismus was classified according to the direction (convergent, divergent, vertical or mixed) and whether it was manifest (present in normal viewing with both eyes open), or latent (present only when the eyes were dissociated and/or one covered). Additional information regarding previous visual difficulties was obtained via a questionnaire.

Appendix B Mediating factors

Intelligence quotient (IQ), attention, social cognition and behaviour were selected as potential mediating factors. Information on these domains was collected during a research clinic to which all ALSPAC participants were invited to attend at age 8. IQ was tested using a shortened form of the Wechsler Intelligence Scale for Children, 3rd edition (WISC-III)(31); this comprised five verbal subtests and five performance subtests from which a total age-scaled score was derived. Selective attention was assessed using the Sky Search Test, taken from the Test of Everyday Attention for children(32). The Diagnostic Analysis of Non-Verbal Accuracy (DANVA) faces subtest was used to test social cognition; we used a binary measure comprising the proportion of children with >=7 errors on the facial expressions recognition task(33). Finally, behaviour was assessed using the Strength and Difficulties Questionnaire (SDQ), completed by parents when children were 9 years of age(34). This comprises questions relating to hyperactivity, emotional symptoms, conduct and peer problems.

Appendix B Multiple imputation for missing data

To minimise the potential bias introduced by missing data, we performed multiple imputation using the mi impute command in Stata v.15.0. The exposure, confounding and mediating factors and outcome variables included in the final model were imputed in the sample of 8,205 that attended the research clinic at 7 years and were eligible for inclusion in the study sample. In addition, factors that predicted missingness were included in the model, thus the assumption of "missing at random" is supported.

Table ST1 summarises the variables and proportion of missing values and compares observed distributions to their distributions after the multivariate imputation for used to impute missing values.

Variable	Number missing (%)	Observed data	Complete data
		% for categorical/binary variables or mean (SD) for continuous variables	
**Hearing impairment** (OME and/or conductive hearing loss)	802 (9.8%)	10.3	10.4
**Visual impairment** (Amblyopia and/or strabismus and/or reduced acuity)	1005 (12.2%)	6.9	7.0
**Maternal age <25 years**	451 (5.5%)	21.6	21.8
**Admission to SCBU**	821 (10.0%)	6.7	6.9
**Ethnicity – white**	860 (10.5%)	96.0	95.9
**Maternal smoking during pregnancy**	1410 (17.2%)	20.0	20.3
**Achievement of ≥ Level 4 at KS2 English**	49 (0.6%)	75.2	75.2
**Achievement of ≥ Level 4 at KS2 Maths**	1050 (12.8%)	82.9	81.9
**Achievement of ≥ Level 4 at KS2 Science**	47 (0.6%)	82.9	83.0
**Achievement of ≥ 5 GCSEs at A*- C** (including Maths & English)	1256 (15.3%)	59.8	56.3
**DANVA** (≥7 mistakes)	2038 (24.8%)	22.3	23.3
**Family Adversity Index (1 – 12)**	643 (7.8%)	1.75 (1.89)	1.75 (1.89)
**Wechsler Intelligence Scale (WISC-III)**	1547 (18.9%)	104.7 (16.3)	104.5 (16.4)
**Strengths & Difficulties Questionnaire (SDQ)**	1703 (20.8%)	6.75 (4.84)	6.77 (4.96)
**Sky Search Test**	1710 (20.8%)	5.20 (1.9)	5.21 (2.0)
**Index of Multiple Deprivations**	1968 (24.0%)		
**1**		33.1	32.2
**2**		19.6	19.3
**3**		18.9	18.9
**4**		15.3	15.8
**5**		13.1	13.8
**Parity**	716 (8.7%)		
**0**		46.1	46.3
**1**		36.0	35.8
**>=2**		17.9	17.9

Table ST1: Proportion of data missing for the variables imputed, and distribution of values in the observed data and after imputation

Confidential: For Review Only

Characteristic	Attended clinic at 7 years (N = 8,283)		Did not attend clinic at 7 years (N = 6,402)		P-value
	N	Percentage of complete data (%)	N	Percentage of complete data (%)	
**Sex**					
Male	4,201	50.7	3,335	52.1	0.09
Female	4,082	49.3	3,066	47.9	
**Low birthweight (<2,500g)**					
Yes	377	4.9	370	6.1	0.00
Missing	549	-	343	-	
**Premature (gestation <37 weeks)**					
Yes	451	5.8	394	6.4	0.11
Missing	455	-	257	-	
**Admission to SCBU**					
Yes	507	6.8	355	7.5	0.17
Missing	830	-	1,644	-	
**Ethnicity**					
White	7,109	95.9	4,359	93.4	0.00
Non-white	301	4.1	308	6.6	
Missing	873	-	1,734	-	
**Parity**					
0	3,477	42.0	2,293	35.8	
1	2,720	32.8	1,819	28.4	0.00
≥2	1,362	16.4	1,253	16.4	
Missing	724	-	1,760	-	
**Maternal age**					
Under 25 years	1,687	21.6	2,602	42.4	0.00
Missing	455	15.6	257	4.0	
**Breastfeeding**					
Yes	3,346	40.4	1,787	27.9	
Missing	4,937	59.6	4,614	72.1	0.00
**Maternal smoking during pregnancy**					
Yes	1,371	20.0	1,529	36.0	0.00
Missing	1,429	-	2,157	-	
**Family Adversity Index at 2-4 years**					
<3	5,646	74.0	3,096	63.3	0.00
≥3	1,987	26.0	1,794	36.7	
Missing	651	-	1,511	-	
**Index of Multiple Deprivations at 7 years**					
1	2,079	33.0	318	23.2	0.00
≥2	4,213	67.0	1,054	76.8	
Missing	1,991	-	5,029	-	
**KS2 ≥ level 4**					
**English**					
Yes	6,172	75.0	3,616	56.9	0.00
Missing	995	-	1,653	-	
**Maths**					
Yes	5,962	82.6	3,536	69.1	0.00
Missing	1,050	-	1,212	-	
**Science**					
Yes	6,810	82.7	4,346	31.7	0.00
Missing	992	-	1,178	-	
**GCSEs ≥5 grade A*-C including Maths & English**					
Yes	4,177	59.5	1,875	37.2	0.00
**SEN at KS2**					
Yes (School action, Action plus or SEN)	948	13.7	1,162	22.8	
Missing	1,379	-	1,307	-	
**SEN at KS4**					
Yes (School action, Action plus or SEN)	733	11.3	859	18.2	
Missing	1,813	-	1,689	-	

Table ST2: Demographic characteristics of children that attended the ALSPAC research clinic at 7 years compared to those that did not attend.

Confidential: For Review Only

Characteristic	Normal Vision & Hearing (n= 2,473)		Hearing Impairment alone (n=247)			Visual Impairment alone (n=175)			Co-occurring hearing & visual Impairment (n=14)		
	N	Percentage (%)	N	Percentage (%)	P-value	N	Percentage (%)	P-value	N	Percentage (%)	P-value
**Sex**											
Male	1,218	49.3	125	50.6	0.68	79	45.1	0.29	10	71.4	0.10
Female	1,255	50.7	122	49.4		96	54.9		<5	28.6	
**Low birthweight (<2,500g)**	89	3.6	10	4.1	0.72	11	6.3	0.07	<5	7.1	0.48
**Premature (gestation <37 weeks)**	121	4.9	16	6.5	0.28	11	6.3	0.41	<5	14.3	0.11
**Admission to SCBU**	134	5.4	23	9.3	0.01	11	6.3	0.62	<5	0	0.37
**Ethnicity**											
White	2,398	97.0	241	97.6	0.60	172	98.3	0.32	14	100	0.51
Non-white	75	3.0	6	2.4		<5	1.7		<5	0	
**Parity**											
0	1,122	45.4	121	49.0	0.28	77	44.0	0.73	6	42.9	0.85
1	920	37.2	79	32.0	0.11	78	44.6	0.05	6	42.9	0.66
≥2	431	17.4	47	19.0	0.53	20	11.4	0.04	<5	14.3	0.76
**Maternal age <25 years**	452	18.3	48	19.4	0.65	39	22.3	0.19	<5	14.3	0.70
**Breastfed**	1,103	44.6	106	42.9	0.61	85	48.6	0.31	<5	28.6	0.23
**Maternal smoking during pregnancy**	445	18.0	39	15.8	0.39	43	24.6	0.03	5	35.7	0.09
**Family Adversity Index at 2-4 years**											
<3	2,089	84.5	205	83.0	0.54	139	79.4	0.08	11	78.6	0.54
≥3	384	15.5	42	17.0		36	20.6		<5	21.4	
**Index of Multiple Deprivations at 7 years**											
1	822	33.2	72	29.2	0.19	54	30.9	0.52	5	35.7	0.84
≥2	1,652	66.8	175	70.9		121	69.1		9	64.2	

Table ST3: Demographic characteristics of children according to their hearing and vision status. NB. "<5" may include zero.

	KS2 English		KS2 Maths		KS2 Science	
	N	Percentage (95% CI)	N	Percentage (95% CI)	N	Percentage (95% CI)
**Normal vision & hearing N=2,473**	2,306	93.3 (92.3 – 94.2)	2,429	98.2 (97.7 – 98.7)	2,421	97.9 (97.3 – 98.5)
**Hearing impairment N= 247**	226	91.5 (88.0 – 95.0)	239	96.8 (94.5 – 99.0)	241	97.6 (95.6 – 99.5)
**Visual impairment N= 175**	149	85.1 (79.8 – 90.5)	161	92.0 (87.9 – 96.1)	165	94.3 (90.8 – 97.8)
**Co-occurring hearing & visual impairment N=14**	10	71.4 (44.4 – 98.5)	12	85.7 (64.8 - >100)	13	92.9 (77.4 - >100)

Table ST4: Proportions of children achieving level 4 or above at Key Stage 2 English, Maths and Science by hearing and vision status

	Normal hearing & vision N = 2480	Hearing impairment N= 247	Visual impairment N= 175	Co-occurring hearing & visual impairment N=14	P-value
**SEN support at KS2** Number Percent (%) (95% CI)	153 6.2 (5.2 – 7.7)	21 8.5 (5.4 – 12.3)	20 11.4 (7.0 – 16.3)	<5 14.3 (<0 – 35.3)	0.02
**SEN support at KS4** Number Percent (%) (95% CI)	147 5.94 (5.0 – 6.9)	17 9.7% (5.4 – 12.3)	21 11.05 (6.6 – 15.6)	<5 28.6 (1.5 – 55.6)	0.01

Table ST5: Proportions of children receiving SEN support at KS2 & KS4 by hearing and vision status

KS2 Maths: Achievement of ≥level 4												
	**Univariate logistic regression**		**Model 1** : Male, age at testing, SEN at KS2		**Model 2:** Model 1 & maternal age <25, parity & smoking during pregnancy		**Model 3:** Model 2 & FAI & IMD		**Model 4:** Model 3 & IQ		**Model 5:** Model 4 & behaviour, social perceptions & attention	
	**OR**	**95% CI**	**OR**	**95% CI**	**OR**	**95% CI**	**OR**	**95% CI**	**OR**	**95% CI**	**OR**	**95% CI**
**Hearing impairment** (n=852)	0.83	0.68 – 1.01	0.90	0.71 – 1.15	0.90	0.71 – 1.15	0.90	0.71 – 1.15	0.91	0.72 – 1.16	0.91	0.71 – 1.15
**Visual impairment** (n=573)	0.66	0.53 – 0.83	0.81	0.61 – 1.07	0.80	0.60 – 1.06	0.81	0.61 – 1.07	0.81	0.61 – 1.08	0.82	0.61 – 1.08
**Co-occurring hearing & visual impairment** (n=45)	0.33	0.17 – 0.63	0.47	0.22 – 1.01	0.46	0.21 – 1.01	0.46	0.21 – 1.01	0.46	0.21 – 1.00	0.46	0.21 – 1.00

Table ST6: Multivariate regression models using the imputed dataset (N=8,205) demonstrating the likelihood children achieved ≥level 4 at KS2 Maths according to hearing and visual status and after adjustment for confounding variables (models 1-3) and mediating factors (models 4-5). OR=Odds Ratio

Confidential: For Review Only

KS2 Science: Achievement of ≥level 4												
	**Univariate logistic regression**		**Model 1** : Male, age at testing, SEN at KS2		**Model 2:** Model 1 & maternal age <25, parity & smoking during pregnancy		**Model 3:** Model 2 & FAI & IMD		**Model 4:** Model 3 & IQ		**Model 5:** Model 4 & behaviour, social perceptions & attention	
	**OR**	**95% CI**	**OR**	**95% CI**	**OR**	**95% CI**	**OR**	**95% CI**	**OR**	**95% CI**	**OR**	**95% CI**
**Hearing impairment** (n=852)	0.89	0.73 – 1.08	0.97	0.77 – 1.22	0.97	0.77 – 1.22	0.97	0.77 – 1.22	0.99	0.78 – 1.24	0.99	0.78 – 1.24
**Visual impairment** (n=573)	0.69	0.54 – 0.87	0.83	0.64 – 1.08	0.84	0.65 – 1.09	0.84	0.65 – 1.10	0.86	0.66 – 1.12	0.87	0.66 – 1.13
**Co-occurring hearing & visual impairment** (n=45)	0.33	0.18 – 0.61	0.46	0.22 – 0.95	0.47	0.23 – 0.97	0.47	0.23 – 0.97	0.48	0.23 – 0.99	0.48	0.23 – 0.99

Table ST7: Multivariate regression models using the imputed dataset (N=8,205) demonstrating the likelihood children achieved ≥level 4 at KS2 Science according to hearing and visual status and after adjustment for confounding variables (models 1-3) and mediating factors (models 4-5). OR=Odds Ratio

Confidential: For Review Only

KS2 English: Achievement of ≥level 4								
	**Univariate logistic regression**		**Model 1** : Male, age at testing, SEN at KS2		**Model 2:** Model 1 & maternal age <25, parity & smoking during pregnancy		**Model 3**: Model 2 & FAI & IMD	
	**OR**	**95% CI**	**OR**	**95% CI**	**OR**	**95% CI**	**OR**	**95% CI**
**Hearing impairment** (n=261)	0.74	0.48 – 1.15	0.81	0.50 – 1.32	0.80	0.49 – 1.31	0.76	0.47 – 1.24
**Visual impairment** (n=189)	0.39	0.30 – 0.60	0.42	0.26 – 0.67	0.42	0.26 – 0.68	0.42	0.26 – 0.68
**Co-occurring hearing & visual impairment** (n=14)	0.20	0.06 – 0.64	0.21	0.06 – 0.81	0.21	0.05 – 0.84	0.21	0.05 – 0.83

KS2 Maths: Achievement of ≥level 4								
	**Univariate logistic regression**		**Model 1** : Male, age at testing, SEN at KS2		**Model 2:** Model 1 & maternal age <25, parity & smoking during pregnancy		**Model 3:** Model 2 & FAI & IMD	
	**OR**	**95% CI**	**OR**	**95% CI**	**OR**	**95% CI**	**OR**	**95% CI**
**Hearing impairment** (n=261)	0.56	0.28 – 1.11	0.64	0.30 – 1.33	0.66	0.31 – 1.40	0.58	0.27 – 1.23
**Visual impairment** (n=189)	0.21	0.12 – 0.38	0.24	0.12 – 0.46	0.21	0.11 – 0.41	0.20	0.10 – 0.41
**Co-occurring hearing & visual impairment** (n=14)	0.14	0.03 – 0.64	0.15	0.02 – 0.91	0.13	0.02 – 0.87	0.10	0.02 – 0.57

KS2 Science: Achievement of ≥level 4								
	**Univariate logistic regression**		**Model 1** : Male, age at testing, SEN at KS2		**Model 2:** Model 1 & maternal age <25, parity & smoking during pregnancy		**Model 3:** Model 2 & FAI & IMD	
	**OR**	**95% CI**	**OR**	**95% CI**	**OR**	**95% CI**	**OR**	**95% CI**
**Hearing impairment** (n=261)	0.87	0.39 – 1.92	1.00	0.44 – 2.28	1.00	0.43 – 2.31	0.89	0.38 – 2.08
**Visual impairment** (n=189)	0.35	0.18 – 0.68	0.46	0.23 – 0.94	0.49	0.23 – 1.03	0.53	0.24 – 1.16
**Co-occurring hearing & visual impairment** (n=14)	0.31	0.04 – 2.42	0.38	0.04 – 3.57	0.49	0.05 – 5.05	0.35	0.04 – 3.21

Table ST8-ST10: Multivariate regression models using complete cases (N=2,909) demonstrating the likelihood children achieved ≥level 4 at KS2 English, Maths & Science according to hearing and visual status and after adjustment for confounding variables (models 1-3). OR=Odds Ratio

Confidential: For Review Only

KS4: Achievement of ≥5 GCSEs at A*-C, including Maths & English										
	**Univariate logistic regression**		**Model 1 :** Male, SCBU admission, SEN at KS4		**Model 2:** Model 1 & maternal age <25, parity & smoking during pregnancy		**Model 3:** Model 2 & FAI & IMD		**Model 4:** Model 3 & KS2 English, Maths & Science performance	
	**OR**	**95% CI**	**OR**	**95% CI**	**OR**	**95% CI**	**OR**	**95% CI**	**OR**	**95% CI**
**Hearing impairment** (n=261)	0.66	0.51 – 0.86	0.69	0.52 – 0.91	0.66	0.50 – 0.88	0.64	0.48 – 0.86	0.64	0.47 – 0.87
**Visual impairment** (n=189)	0.91	0.66 – 1.26	1.00	0.71 – 1.41	1.08	0.76 – 1.53	1.12	0.77 – 1.59	1.47	0.98 – 2.21
**Co-occurring hearing & visual impairment** (n=14)	0.51	0.18 – 1.49	0.79	0.25 – 2.53	0.82	0.24 – 2.79	0.77	0.22 – 2.64	1.18	0.29 – 4.74

Table ST11: Multivariate regression models using complete cases (N=2,909) demonstrating the likelihood children achieved ≥level 4 at KS2 English, Maths & Science and ≥5 GCSEs at A-C grade (including Maths & English), according to hearing and visual status and after adjustment for. OR=Odds Ratio*

Confidential: For Review Only

BMJ Paediatrics Open

THE IMPACT OF CO-OCCURING HEARING AND VISUAL DIFFICULTIES IN CHILDHOOD ON EDUCATIONAL OUTCOMES: A LONGITUDINAL COHORT STUDY

Journal:	*BMJ Paediatrics Open*
Manuscript ID	bmjpo-2018-000389.R1
Article Type:	Original article
Date Submitted by the Author:	20-Dec-2018
Complete List of Authors:	Hill, Matilda; University of Bristol Centre for Child and Adolescent Health<br>Hall, Amanda; Aston University School of Life and Health Sciences<br>Williams, Cathy; University of Bristol Centre for Child and Adolescent Health<br>Emond, Alan; University of Bristol, Centre for Child and Adolescent Health
Keywords:	Comm Child Health, Ophthalmology, Audiology, Epidemiology, Paediatric Practice

SCHOLARONE™
Manuscripts

THE IMPACT OF CO-OCCURING HEARING AND VISUAL DIFFICULTIES IN CHILDHOOD ON EDUCATIONAL OUTCOMES: A LONGITUDINAL COHORT STUDY

Matilda Hill[1], Amanda Hall[2], Cathy Williams[1], Alan Emond[1]

matildahill84@gmail.com, a.hall@aston.ac.uk, cathy.williams@bristol.ac.uk, alan.emond@bristol.ac.uk

Correspondence to: Dr Matilda Hill, Centre for Academic Child Health, Bristol Medical School, University of Bristol BS8 1NU

Word Count: 3,739

Confidential: For Review Only

[1] Centre for Academic Child Health, Bristol Medical School, University of Bristol BS8 1NU
[2] Life and Health Sciences, Aston University, Birmingham B4 7ET

Article Summary

What is known about the subject

- Mild hearing and visual difficulties are common in childhood.
- The most common cause of hearing difficulties in childhood is chronic otitis media with effusion (OME), which may be associated with lower academic performance.
- The impact of common co-occurring hearing and visual difficulties on educational outcomes is not known.

What this study adds

- Children with co-occurring mild hearing and visual difficulties have poorer educational outcomes than children with isolated hearing or visual difficulties.
- Mild visual difficulties alone do not negatively impact on academic performance at school.

Confidential: For Review Only

Abstract

Background: Mild hearing and visual difficulties are common in childhood, and both may have implications for educational achievement. However, the impact of co-occurring common hearing and visual difficulties in childhood is not known.

Objective: To determine the prevalence and impact of co-occurring common hearing and visual difficulties of childhood on educational outcomes in primary and secondary school.

Methods: The sample was drawn from the Avon Longitudinal Study of Parents and Children (ALSPAC), a longitudinal birth cohort study in England. The exposures were hearing and visual difficulties at age 7 (defined as conductive hearing loss or OME, and amblyopia, strabismus or reduced visual acuity, respectively). The outcomes measured were achievement of level 4 or above at Key Stage 2 (KS2) in English, Maths and Science, respectively, at age 11, and attainment of 5 or more GCSEs at grades A*-C at age 16. Multiple logistic regression models assessed the relationship between hearing and visual difficulties and educational outcomes, adjusting for potential confounding factors.

Results: Children with co-occurring hearing and visual difficulties were less likely to achieve the national target at KS2 compared to children with normal hearing and vision, even after adjustment for confounding factors. Differences in IQ, behaviour, attention and social cognition did not account for this relationship. The impact of co-occurring hearing and visual difficulties on GCSE results was explained largely by poor performance at KS2.

Conclusions: Co-occurring hearing and visual difficulties in childhood have an enduring negative impact on educational outcomes. Identification of affected children and early intervention in primary school is essential.

Confidential: For Review Only

Confidential: For Review Only

Introduction

Hearing and visual difficulties are prevalent amongst children in the UK. The majority of affected children have mild, temporary difficulties, which may not be formally diagnosed and for which they do not receive additional educational support. However, there is evidence that even mild deficits in hearing or vision can have implications for learning and development throughout childhood(1-5).

The most common cause of chronic hearing loss in childhood is persistent otitis media with effusion (OME, ‘glue ear’). There is ongoing controversy regarding the impact of OME on language development and cognition. Whilst several large studies have found no association between OME and language or academic attainment(6, 7), few of these studies have considered concurrent hearing loss or chronicity of OME. Conversely, a number of studies have demonstrated an association between persistent OME-related hearing loss, cognitive development, reading ability and behavioural problems (2-4, 8).

The impact of common visual difficulties on academic attainment in childhood is not well characterised(9). Decreased visual acuity in childhood is associated with reduced literacy(1), but the consequences of more common visual difficulties are not clear. Amblyopia and strabismus are two of the most common visual difficulties encountered in childhood(10), which often occur in the context of conditions which may in themselves affect development and educational achievement such as prematurity and low birthweight(11, 12), thus rigorous adjustment for confounding factors is required to establish the functional impact of these visual difficulties in of themselves.

The impact of co-occurring common hearing and visual difficulties on educational outcomes in childhood has not previously been studied. However, it is well-established that integration of auditory and visual information is essential for many cognitive processes, in particular speech perception, and key for the development of language and communication skills(13, 14). A deficit in one of these

Confidential: For Review Only

domains in childhood may therefore hinder developing literacy and render an individual vulnerable to the cumulative effect of an additional sensory deficit.

The aim of this study is to investigate the prevalence and impact of co-occurring common hearing and visual difficulties in childhood on educational outcomes at primary school and secondary school using a large population cohort. We hypothesise that children with co-occurring mild hearing and visual difficulties are more likely to have poorer language and communication skills, and thus lower levels of academic attainment, relative to those with a single sensory deficit or children with normal hearing and vision.

Methods

Study Participants

The Avon Longitudinal Study of Parents and Children (ALSPAC) is a longitudinal population-based birth cohort study. All pregnant women resident in Avon, UK, with expected dates of delivery between 1st April 1991 and 31st December 1992 were eligible for participation, resulting in enrolment of over 14,000 live births(15, 16). Ethical approval for the study was obtained from the ALSPAC Ethics and Law Committee and the Local Research Ethics Committees.

Please note that the study website contains details of all the data that is available through a fully searchable data dictionary and variable search tool: http://www.bristol.ac.uk/alspac/researchers/our-data/

Patient and Public Involvement

ALSPAC participants advise on ALSPAC studies through the original cohort advisory panel (OCAP) and contribute to the ALSPAC ethics and law committee. ALSPAC participants were not directly involved in the design of this study.

Hearing and vision assessments

Confidential: For Review Only

All participating children in ALSPAC were invited to attend a research clinic at 7 years of age. 59.3% (8,299 children) attended during the period September 1998 - September 2000, of whom 98.9% (8,205 children) were eligible for inclusion (see Figure 1). The clinics included a comprehensive assessment of vision and hearing, details of which are included in Appendix A of the Supplementary Material.

Definition of Hearing Difficulties

Hearing difficulties were defined as the presence of mild-moderate conductive hearing loss and/or OME in either ear, characterised by air conduction greater than 20 dB and less than 70 dB averaged across 500 Hz, 1kHz, 2kHz and 4kHz (based on The British Society of Audiology definitions(17)), or the presence of a type B tympanogram, respectively. The prevalence of OME decreases with age after the first 2 years of life(18); children with evidence of OME at age 7 are likely to have persistent OME which is associated with conductive hearing loss in over 70% of cases(19, 20). However, hearing loss may be fluctuant and thus not captured by a single clinic assessment, hence the utilisation of both air conduction tests and tympanometry to identify children with hearing difficulties.

A total of 5 children with sensorineural hearing loss, defined by bone conduction greater than 30 dB at either 1 kHz or 4 kHz, were excluded from this analysis.

Definition of Visual Difficulties

Children with "clinically significant" strabismus, amblyopia or mild-moderate reduced acuity (based on the WHO ICD 11 definition(21)) were defined as having visual difficulties. "Clinically significant" strabismus comprised all children with manifest strabismus, or previously defined large latent deviations (>=10 pd if convergent & >=15pd if divergent)(10). Amblyopia was defined as a history of patching treatment and/or an interocular difference in acuity of >0.2logMAR units where the worst-seeing eye had an acuity of >0.3logMAR. Reduced acuity was defined by reduced distance acuity of the better-seeing eye >=0.3 logMAR. Acuity was assessed with glasses if worn ('habitual' state), and in the 'habitual state plus pinhole', as a proxy for full refractive correction.

Confidential: For Review Only

Refractive errors were not included in our definition of visual difficulties as these are potentially correctable with glasses and have already been studied extensively in the context of educational achievement, with myopia being linked with higher educational achievement(22, 23).

19 children with known ocular pathology or severe visual impairments (>1.0logMAR) were excluded. Triplets, quadruplets and children with Down's Syndrome or Cerebral Palsy were also excluded from this analysis.

Educational Outcomes

Educational outcomes at primary school were assessed using Standardised Assessment Test (SAT) results, obtained from the National Pupil Database (NPD)(24). Key Stage 2 (KS2) tests are undertaken during the final year of primary school (year 6) at age 10-11. The national expected standard is achievement of National Curriculum level 4 or above, and we therefore used achievement of ≥level 4 at KS2 in English, Maths and Science as our three educational outcomes in primary school; performance in each subject was analysed separately.

Educational outcomes in secondary school were assessed using General Certificate of Secondary Education (GCSE) results at Key Stage 4 (KS4). GCSEs are taken at the end of compulsory schooling (year 11) at age 16, and are graded A*- G. Achievement of 5 or more GCSEs (including Maths and English) at grades A*- C is the national benchmark measure of achievement, and we used this as our single outcome at secondary school. We adjusted outcomes for KS2 attainment, as performance at KS2 is well known to predict performance at GCSEs(25).

Information on children receiving Special Educational Needs (SEN) support was provided by the Pupil Level Annual School Census (PLASC). SEN status was obtained for each child at KS2 and KS4, with information on the level of individual educational support being provided. We

Confidential: For Review Only

dichotomised these data into children receiving no special provision and those receiving some level of support.

NPD and PLASC data were not available for children attending independent schools, or schools outside of England, and these children were excluded from this analysis.

Confounding and Mediating Factors

Gender, prematurity, low birthweight, admission to a Special Care Baby Unit (SCBU), maternal age, parity, smoking during pregnancy, duration of breastfeeding and socioeconomic status were selected as potential confounding factors, based on their previously identified associations with hearing or visual difficulties in childhood and well-established links with academic performance(10-12, 26-29). The ALSPAC Family Adversity Index (FAI), derived from a questionnaire about socioeconomic status completed by mothers at 2-4 years, was used as a measure of family adversity. The Indices of Multiple Deprivations (IMD) at age 7, a census-derived score of relative deprivation of a neighbourhood was used as a measure of social deprivation.

Intelligence quotient (IQ), attention, social cognition and behaviour were selected as potential mediating factors, as they may be influenced by visual and hearing difficulties and can impact on educational achievement(1, 3, 4, 8). Reading ability was not included due to high rates of missing data. These domains were tested during a research clinic to which all participating children were invited and 7,488 children (53.5%) attended at 8 years of age. Further information on these assessments is provided in Appendix B of the Supplementary Material.

Analyses

Binary univariate logistic regressions were used to calculate odds ratios (OR) and 95% confidence intervals (95% CI) to assess the relationship between hearing and visual difficulties and educational outcomes. The analyses were repeated, controlling for all potential confounding factors significant at the 5% level in the univariate analyses and all potential mediating factors which fulfilled the Baron

Confidential: For Review Only

and Kenny mediation model steps 1-3(30). These multiple logistic regression models sequentially adjusted for individual factors, maternal factors, wider socioeconomic factors, earlier educational performance, IQ and additional mediating factors. Further information is provided in Tables 2 & 3.

Multiple imputation using chained equations (ICE) was used to impute missing data for all variables included in the final logistic regression models, including the outcome, and variables that predicted missingness. This technique helps to minimise attrition bias and improve precision of estimates(31). We imputed data for all 8,205 children who attended the research clinic at 7 years and did not meet the exclusion criteria. Twenty imputations were performed. All analyses were carried out using STATA version 15.0 (StataCorp 2017). Further information regarding the multiple imputation is provided in Appendix C of the Supplementary Material.

Confidential: For Review Only

Results

Sample Characteristics

Of the 8,205 children that attended the research clinic at 7 years and were eligible for inclusion, 2,909 (33.5%) had complete data for hearing and vision, educational outcomes and potential confounding variables (see Figure 1).

Children attending the research clinic were more likely to be come from families of higher socioeconomic class and achieve higher levels of academic attainment at both KS2 and KS4 than children that did not attend the clinic (see supplementary material, Table ST2).

Prevalence and Causes of Hearing & Visual Difficulties

Of the 2,909 children with complete data, 261 (9.0%) had hearing difficulties, 189 (6.5%) had visual difficulties and 14 (0.5%) had both hearing and visual difficulties. Types of hearing and visual difficulties are demonstrated in Table 1.

Conductive hearing loss was identified in 159 children; 145 of these children (91%) had mild hearing loss (air conduction of 21.25 – 40 dB, mean 27.68dB), and 14 children had moderate hearing loss (air conduction 41.25dB – 66.25dB, mean 46.25dB). The majority of children with conductive hearing loss had evidence of concurrent OME.

The overall prevalence of OME in either or both ears was 6.7%. A higher proportion of children undergoing assessment in winter months had OME compared to those attending clinic in the summer months (8.0% vs 5.6%), but this association was weak (p=0.09).

**Types of Hearing and Visual Difficulties**	**Proportion of children with either hearing or visual difficulties (%)**
**Hearing Difficulties**	**261 (100%)**
Otitis Media with Effusion	102 (39.1%)
Otitis Media with Effusion & Conductive Hearing Loss	94 (36.0%)
Conductive Hearing Loss	65 (24.9%)
**Visual Difficulties**	**189 (100%)**
Amblyopia	79 (41.8%)
Strabismus	56 (29.6%)
Strabismus & Amblyopia	47 (24.9%)

Confidential: For Review Only

Reduced Acuity with or without Amblyopia and/or Strabismus	7 (3.7%)

Table 1: Types of hearing and visual difficulties

Confidential: For Review Only

Amblyopia was the most common cause of visual difficulties, affecting 4.4% of all children. There was considerable overlap between amblyopia and strabismus; of the 128 children with amblyopia, 47 (36.7%) also had strabismus.

Only 7 children had reduced habitual acuity in the best-seeing eye; 4 children had mild reduced visual acuity (0.3logMAR – 0.44logMAR), and 3 children had moderate reduced acuity (0.50logMAR – 0.7logMAR), with a mean acuity of 0.45logMAR. These reduced acuities most likely represent uncorrected refractive errors, given that acuity was not assessed with full refractive correction and the exclusion of children with ocular pathology.

Children with hearing and visual difficulties had similar characteristics to those with normal hearing and vision (see supplementary material, Table ST3). However, children with visual difficulties were more likely to have mothers who smoked during pregnancy (24.6% vs 18.0%, p=0.03) and children with hearing difficulties were more likely to have been admitted to SCBU in infancy (9.3% vs 5.4%, p=0.01).

Educational Outcomes and SEN status in Primary School

The proportion of children in this study achieving level 4 or above at KS2 exceeded the national average; 92.5% and 97.7% of children achieved level 4 or above in KS2 English and Maths tests respectively, compared to 75% and 73% of children nationally(32). The proportions of children achieving these targets by hearing and visual status are shown in supplementary material, Table ST4.

Children with either hearing or visual difficulties were less likely to achieve level 4 or above at KS2 English tests relative to children with normal hearing and vision (see Table 2 for the imputed dataset analysis; see supplementary material, Table ST8-ST10 for the complete cases analysis). However, these relationships were attenuated by adjustment for confounding factors, and further attenuated after adjustment for IQ, suggesting that some of the association is mediated through IQ. In contrast, children with co-occurring hearing and visual difficulties remained less likely to achieve this target after controlling for confounding factors (OR 0.30, CI 0.15-0.61). This relationship was not significantly altered by adjustment for IQ or other potential mediating factors.

Confidential: For Review Only

KS2 English: Achievement of ≥level 4												
	**Univariate logistic regression**		**Model 1** : Male, age at testing, SEN at KS2		**Model 2:** Model 1 & maternal age <25, parity & smoking during pregnancy		**Model 3:** Model 2 & FAI & IMD		**Model 4:** Model 3 & IQ		**Model 5:** Model 4 & behaviour, social perceptions & attention	
	**OR**	**95% CI**	**OR**	**95% CI**	**OR**	**95% CI**	**OR**	**95% CI**	**OR**	**95% CI**	**OR**	**95% CI**
**Hearing difficulties** (n=852)	0.78	0.66 – 0.92	0.80	0.66 – 0.98	0.81	0.66 – 0.99	0.81	0.66 – 0.99	0.87	0.71 – 1.06	0.88	0.72 – 1.08

**Visual difficulties** (n=573)	0.66	0.64 – 0.80	0.79	0.62 – 0.99	0.81	0.64 – 1.02	0.82	0.64 – 1.03	0.87	0.68 – 1.11	0.90	0.71 – 1.14
**Co-occurring hearing & visual difficulties** (n=45)	0.25	0.14 – 0.45	0.30	0.15 – 0.61	0.30	0.15 – 0.61	0.30	0.15 – 0.61	0.36	0.17 – 0.74	0.38	0.18 – 0.78

Table 2: Multiple regression models using the imputed dataset (N=8,205) demonstrating the likelihood children achieved ≥level 4 at KS2 English according to hearing and visual status and after adjustment for confounding variables (models 1-3) and mediating factors (models 4-5). OR=Odds Ratio

The relationship is similar for performance in KS2 Maths and KS2 Science tests, albeit hearing difficulties appear to have less of an impact (see supplementary material, Table ST6 and ST7).

The proportion of children with visual difficulties receiving SEN support was higher compared to children with normal hearing and vision (11.4% vs 6.2%, p =0.02, see supplementary material, Table ST5). Overall, children with SEN status at KS2 were less likely to achieve level 4 or above in KS2 English, Maths and Science tests than those without formal support (52.6% vs 87.6%, p=0.00).

Educational Outcomes and SEN Status in Secondary School

Overall, almost three-quarters (72.1%) of the study sample achieved 5 or more GCSEs (including English and Maths) at A*-C grade, which is significantly higher than the national average of 47.6% children achieving this standard the same year(33). The proportion of children with hearing difficulties achieving this target was lower compared to those with normal hearing and vision (see Figure 2).

KS4: Achievement of ≥5 GCSEs at A*-C, including Maths & English

Confidential: For Review Only

	**Univariate logistic regression**		**Model 1 :** Male, SCBU admission, SEN at KS4		**Model 2:** Model 1 & maternal age <25, parity & smoking during pregnancy		**Model 3:** Model 2 & FAI & IMD		**Model 4:** Model 3 & KS2 English, Maths & Science performance		**Model 5:** Model 4 & IQ		**Model 6**: Model 5 & behaviour, social perceptions & attention	
	**OR**	**95% CI**	**OR**	**95% CI**	**OR**	**95% CI**	**OR**	**95% CI**	**OR**	**95% CI**	**OR**	**95% CI**	**OR**	**95% CI**
**Hearing difficulties** (n=852)	0.80	0.68 – 0.94	0.83	0.69 – 0.99	0.83	0.69 – 0.99	0.82	0.68 – 0.99	0.85	0.69 – 1.04	0.95	0.76 – 1.17	0.96	0.77 – 1.19
**Visual difficulties** (n=573)	0.86	0.71 – 1.05	0.94	0.74 – 1.17	1.00	0.79 – 1.27	1.02	0.81 – 1.30	1.20	0.92 – 1.56	1.34	1.00 – 1.79	1.37	1.02 – 1.84
**Co-occurring hearing & visual difficulties** (n=45)	0.55	0.29 – 1.06	0.67	0.29 – 1.51	0.68	0.29 – 1.58	0.66	0.28 – 1.53	1.13	0.43 – 2.94	1.42	0.50 – 4.06	1.49	0.52 – 4.29

Table 3: Multiple regression models using the imputed dataset (N=8,205) demonstrating the likelihood children achieved ≥5 GCSEs at A-C grade (including Maths & English), according to hearing and visual status and after adjustment for confounding variables (models 1-3) and mediating factors (models 4-5). OR=Odds Ratio*

The association between hearing difficulties and poorer performance at GCSEs is attenuated after adjustment for performance at KS2 and IQ, suggesting that the association between hearing and education is mediated through these factors (see Table 3 for the imputed dataset analysis; see supplementary material, Table ST11 for the complete cases analysis). Children with visual difficulties alone were no less likely to attain 5 or more GCSEs at A*-C; they were in fact more likely to achieve this target after adjustment for confounding variables and IQ.

Children with co-occurring hearing and visual difficulties were less likely to achieve the national target even after adjustment for confounding factors, although this association was weak (OR 0.66, CI 0.28-1.53). This relationship is attenuated after adjustment for performance at KS2, suggesting that

Confidential: For Review Only

poorer outcomes at KS4 in these children can be partly explained by poorer educational outcomes at primary school.

At KS4, the overall proportion of children receiving SEN support was similar to that at KS2 (6.7% vs 6.5%, p=0.76). Children with visual difficulties were almost twice as likely to receive SEN support than those with normal hearing and vision (see supplementary material, Table ST5). Children with SEN provision were less likely to attain 5 GCSEs at A*-C (32.3% vs 74.9%, p=0.00).

Confidential: For Review Only

Discussion

Discussion of results

In this longitudinal study we have demonstrated that co-occurring mild hearing and visual difficulties in childhood have a negative impact on educational outcomes, greater than the effect of hearing or visual difficulties alone. We have shown a weak association between mild hearing difficulties at age 7 and academic achievement at KS2 and KS4, which may be mediated by IQ. We found no negative association between mild visual difficulties and academic outcomes. In contrast, children with co-occurring hearing and visual difficulties were less likely to attain the expected academic standards at the end of primary school, an effect which is not wholly mediated through IQ, behaviour, attention or social cognition, suggesting a substantial, unexplained educational disadvantage. They were also less likely to achieve 5 or more GCSEs at Key Stage 4, which may be attributable to poor performance at KS2. However, this relationship is weak, likely due to the relatively low prevalence of co-occurring mild sensory difficulties and potential small effect size.

A higher proportion of children with visual difficulties had SEN status at both primary school and secondary school relative to those with normal hearing and vision. Common visual difficulties, as defined by this study, are of themselves not sufficient to merit SEN support, suggesting a higher prevalence of additional disabilities amongst children with visual difficulties. This is consistent with

previously reported findings(34). Children receiving SEN support are less likely to achieve the national targets at both primary school and secondary school compared to those without SEN support, implying SEN status is a proxy indicator of severity of educational difficulties.

Strengths and Weaknesses

The major strength of this study is that it uses a large population-based birth cohort, with an accurate assessment of hearing and vision. Furthermore, it uses standardised national tests as objective outcome measures, and longitudinally collected data on a wide range of confounders.

The most important limitation of this study relates to missing data and the under-representation of children from ethnic minorities and those from lower socio-economic backgrounds. A multiple imputation technique was utilised to minimise potential bias and improve precision. An additional limitation is the single time-point used to assess hearing, and possible inclusion of children with transient OME as a result, which may have led to underestimation of the effect of persistent OME-related hearing loss on academic attainment. Furthermore, we did not have information regarding which children were treated for their hearing and visual difficulties either prior or following the assessment at age 7. Finally, whilst we excluded children with known diagnoses of Down's Syndrome or Cerebral Palsy, we did not have full data regarding developmental disorders or medical co-morbidities. However, such conditions affect only a small minority of children with hearing or visual difficulties and this was a population-based study designed to evaluate the impact of common, mild difficulties(12, 35).

In the context of other research

Previous studies investigating the impact of co-occurring hearing and visual difficulties in childhood have focused on children with severe deficits. Developmental disorders commonly co-occur with severe hearing and visual difficulties, or 'deaf-blindness'(34-36), which often precludes children from attending mainstream schools and has additional implications for their education. To our knowledge,

Confidential: For Review Only

our study is the first to investigate the impact of co-occurring common mild hearing and visual difficulties upon academic attainment, in a population of children attending mainstream schools.

The impact of childhood OME on cognition and educational performance has been studied previously by prospective cohort studies, although there is no clear consensus. Data from the longitudinal Dunedin study demonstrated an association between childhood OME and lower IQ in early teenage years(2), which is consistent with findings from the Aarhus Birth Cohort that reported hearing loss at 9 -11 years was associated with behavioural problems and reading difficulties(8). In contrast, data from the Danish National Birth Cohort showed no association between childhood OME episodes and school performance, findings supported by a meta-analysis of prospective studies, which found little to no association between OME and speech and language development(6, 7). The disparity in these findings is likely explained by the heterogeneity in study design, including inconsistencies in adjustment for confounding variables and consideration of associated hearing loss and persistence of OME. Our findings demonstrated a small effect of OME and/or hearing loss at age 7 on academic performance at age 10-11 and 16 years after adjustment for confounding variables, mediated through IQ. This is consistent with data from the 1970 British Birth Cohort(3), and previously published work using ALSPAC data, which have shown an association between OME and lower IQ(4), although their results suggest this association diminishes with age.

Previous research investigating the impact of common visual difficulties in childhood on educational outcomes has focused largely on refractive errors, which were not included in this study(22, 23). There is, however, convincing evidence from a cross-sectional study involving participants of the Born in Bradford birth cohort study that reduced visual acuity at age 4-5 years is associated with reduced literacy(1). However, the prevalence of reduced visual acuity was considerably lower in our study (<1% vs 4%, likely due to the different socioeconomic and ethnic demographics of the cohorts). Data from the 1958 British Birth Cohort demonstrated no impact of unilateral amblyopia in childhood on educational tests at age 7, 11 and 16, or on highest educational qualification obtained(37).

Confidential: For Review Only

Amblyopia was the most common cause of visual difficulties in our study, and our findings are consistent with this.

Furthermore, we demonstrated higher rates of SEN amongst children with visual difficulties; this is consistent with previous research demonstrating that visual difficulties commonly co-occur with developmental disorders(35). We are, however, unable to explain the observed positive effect of visual difficulties on performance at secondary school after adjustment for confounders and IQ; a finding that has been reported in the context of refractive errors previously(22).

Clinical and Research Implications

This study has important clinical implications. Children with known visual or hearing difficulties should be routinely tested for additional sensory difficulties, as even mild co-occurring visual or hearing deficits are associated with poorer educational outcomes. The impact of co-occurring hearing and visual difficulties on performance at secondary school is largely explained by poor performance at primary school, hence early identification and intervention is essential.

Future research involving larger numbers of participants are required to replicate these findings and elucidate further the factors mediating this association. We recommend that future investigators focus next on the role of reading and language skills as potential mediators which could explain these findings.

Acknowledgements

We are extremely grateful to all the families who took part in this study, the midwives for their help in recruiting them, and the whole ALSPAC team, which includes interviewers, computer and laboratory technicians, clerical workers, research scientists, volunteers, managers, receptionists and nurses. The authors thank Dr Linda Hollen for providing advice on the statistical methodology.

Funding Statement

Confidential: For Review Only

The UK Medical Research Council, the Wellcome Trust (Grant ref: 102215/2/13/2) and the University of Bristol provide core support for ALSPAC. A comprehensive list of grants funding is available on the ALSPAC website: http://www.bristol.ac.uk/alspac/external/documents/grant-acknowledgements.pdf

Author Contributors

MH, AH, CW & AE designed the study. MH carried out the data analysis & wrote the initial manuscript draft. AH, CW & AE contributed to the analysis, and reviewed and revised the manuscript. The authors declare that there is no conflict of interest in the publication of this research. This publication is the work of the authors, who will serve as guarantors for the contents of this paper.

References

1. Bruce A, Fairley L, Chambers B, Wright J, Sheldon TA. Impact of visual acuity on developing literacy at age 4-5 years: a cohort-nested cross-sectional study. BMJ Open. 2016;6(2):e010434.
2. Bennett KE, Haggard MP, Silva PA, Stewart IA. Behaviour and developmental effects of otitis media with effusion into the teens. Archives of disease in childhood. 2001;85(2):91-5.
3. Bennett KE, Haggard MP. Behaviour and cognitive outcomes from middle ear disease. Archives of disease in childhood. 1999;80(1):28-35.
4. Hall AJ, Maw R, Midgley E, Golding J, Steer C. Glue ear, hearing loss and IQ: an association moderated by the child's home environment. PloS one. 2014;9(2):e87021.
5. Archbold S YZ, Harrigan S Gregory S, Wakefield t, Holland L, Mulla I. Experiences of young people with mild to moderate hearing loss. National Deaf Children's Society; 2015.
6. Fougner V, Korvel-Hanquist A, Koch A, Dammeyer J, Niclasen J, Lous J, et al. Early childhood otitis media and later school performance - A prospective cohort study of associations. Int J Pediatr Otorhinolaryngol. 2017;94:87-94.
7. Roberts JE, Rosenfeld RM, Zeisel SA. Otitis media and speech and language: a meta-analysis of prospective studies. Pediatrics. 2004;113(3 Pt 1):e238-48.
8. Niclasen J, Obel C, Guldager C, Pleinert S, Dammeyer J. Associations between childhood hearing loss and behavioural and academic difficulties: A Danish cohort study. Int J Pediatr Otorhinolaryngol. 2016;90:91-8.
9. Carlton J, Karnon J, Czoski-Murray C, Smith KJ, Marr J. The clinical effectiveness and cost-effectiveness of screening programmes for amblyopia and strabismus in children up to

Confidential: For Review Only

the age of 4-5 years: a systematic review and economic evaluation. Health technology assessment (Winchester, England). 2008;12(25):iii, xi-194.
10. Williams C, Northstone K, Howard M, Harvey I, Harrad RA, Sparrow JM. Prevalence and risk factors for common vision problems in children: data from the ALSPAC study. Br J Ophthalmol. 2008;92(7):959-64.
11. Robaei D, Kifley A, Gole GA, Mitchell P. The impact of modest prematurity on visual function at age 6 years: findings from a population-based study. Archives of ophthalmology (Chicago, Ill : 1960). 2006;124(6):871-7.
12. Pathai S, Cumberland PM, Rahi JS. Prevalence of and early-life influences on childhood strabismus: findings from the Millennium Cohort Study. Archives of pediatrics & adolescent medicine. 2010;164(3):250-7.
13. Erdener D, Burnham D. The relationship between auditory-visual speech perception and language-specific speech perception at the onset of reading instruction in English-speaking children. Journal of experimental child psychology. 2013;116(2):120-38.
14. McGurk H, MacDonald J. Hearing lips and seeing voices. Nature. 1976;264(5588):746-8.
15. Boyd A, Golding J, Macleod J, Lawlor DA, Fraser A, Henderson J, et al. Cohort Profile: the 'children of the 90s'--the index offspring of the Avon Longitudinal Study of Parents and Children. Int J Epidemiol. 2013;42(1):111-27.
16. Fraser A M-WC, Tilling K, Boyd A, Golding J, Davey Smith G, Henderson J, Macleod J,, Molloy L NA, Ring S, Nelson SM, Lawlor DA. Cohort Profile: The Avon Longitudinal Study of Parents and Children: ALSPAC mothers cohort. International Journal of Epidemiology. 2013;42:97-110.
17. Recommended Procedure : Pure-tone air-conduction and bone-conduction threshold audimetry with and without masking. The British Society of Audiology; 2011.
18. Midgley EJ, Dewey C, Pryce K, Maw AR. The frequency of otitis media with effusion in British pre-school children: a guide for treatment. ALSPAC Study Team. Clinical otolaryngology and allied sciences. 2000;25(6):485-91.
19. Daly KA, Hunter LL, Giebink GS. Chronic otitis media with effusion. Pediatrics in review. 1999;20(3):85-93.
20. Bluestone CD. Otitis Media in Infants and Children. 4th ed: People's Medical Publishing House--USA LTD; 2006.
21. ICD-11 Internation Classification of Disease for Mortality and Morbidity Statistics. World Health Organisation; 2018.
22. Saw SM, Tan SB, Fung D, Chia KS, Koh D, Tan DT, et al. IQ and the association with myopia in children. Invest Ophthalmol Vis Sci. 2004;45(9):2943-8.
23. Mutti DO, Mitchell GL, Moeschberger ML, Jones LA, Zadnik K. Parental myopia, near work, school achievement, and children's refractive error. Invest Ophthalmol Vis Sci. 2002;43(12):3633-40.
24. Education TDf. National Pupil Database, UK data archive: Colchester, Essex.
25. Benton T ST. Analysis of use of Key Stage 2 data in GCSE predictions. ARD Research Division; 2014.
26. Paradise JL, Rockette HE, Colborn DK, Bernard BS, Smith CG, Kurs-Lasky M, et al. Otitis media in 2253 Pittsburgh-area infants: prevalence and risk factors during the first two years of life. Pediatrics. 1997;99(3):318-33.

Confidential: For Review Only

27. Cumberland PM, Pathai S, Rahi JS, Millennium Cohort Study Child Health G. Prevalence of eye disease in early childhood and associated factors: findings from the millennium cohort study. Ophthalmology. 2010;117(11):2184-90 e1-3.
28. Sirin SR. Socioeconomic Status and Academic Achievement: A Meta-Analytic Review of Research. Review of Educational Research. 2005;75(3):417-53.
29. Bowatte G, Tham R, Allen KJ, Tan DJ, Lau M, Dai X, et al. Breastfeeding and childhood acute otitis media: a systematic review and meta-analysis. Acta Paediatr. 2015;104(467):85-95.
30. Baron RM, & Kenny, D. A. The moderator–mediator variable distinction in social psychological research: Conceptual, strategic, and statistical considerations. Journal of Personality and Social Psychology. 1986;51(6):1173-82.
31. Sterne JA, White IR, Carlin JB, Spratt M, Royston P, Kenward MG, et al. Multiple imputation for missing data in epidemiological and clinical research: potential and pitfalls. Bmj. 2009;338:b2393.
32. National Curriculum Assessments at Key Stage 2 in England 2008 (Revised). England: Department for Children, Schools andFfamilies; 2009.
33. GCSE and Equivalent Examination Results in England 2007/08 (Revised). Department for Children, Schools and Families; 2009.
34. Salt A, Sargent J. Common visual problems in children with disability. Archives of disease in childhood. 2014;99(12):1163-8.
35. Kancherla V, Van Naarden Braun K, Yeargin-Allsopp M. Childhood vision impairment, hearing loss and co-occurring autism spectrum disorder. Disabil Health J. 2013;6(4):333-42.
36. NDCS. Prevalence of additional disabilities with deafness: A review of the literature. 2012.
37. Rahi JS, Cumberland PM, Peckham CS. Does amblyopia affect educational, health, and social outcomes? Findings from 1958 British birth cohort. BMJ. 2006;332(7545):820-5.

Figure Legends

Figure 1: Study sample flow chart showing inclusion & exclusion criteria

Figure 2: Graph showing proportion of students achieving ≥5 GCSEs at A-C (including Maths & English) by hearing and vision status*

Confidential: For Review Only

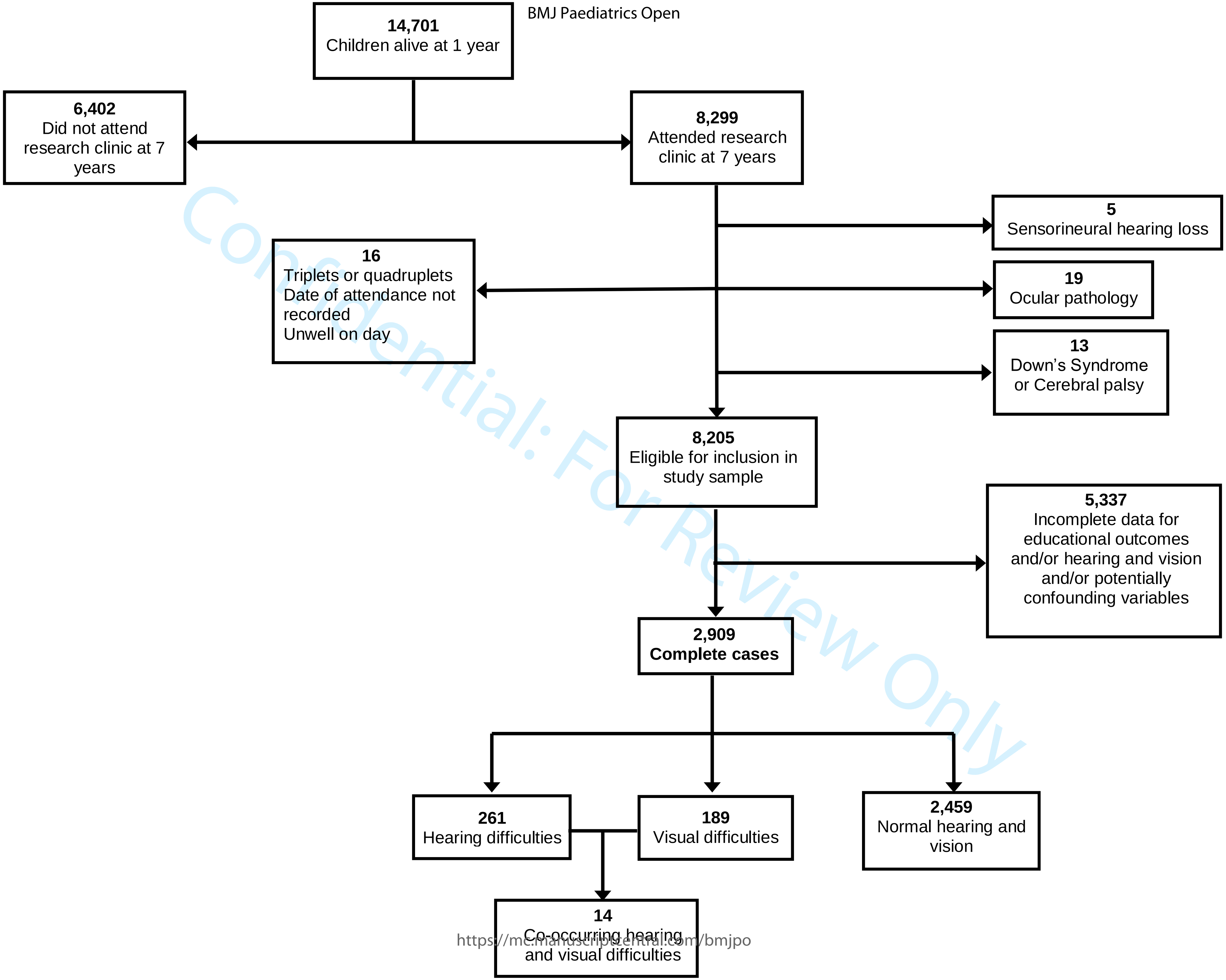

Confidential: For Review Only

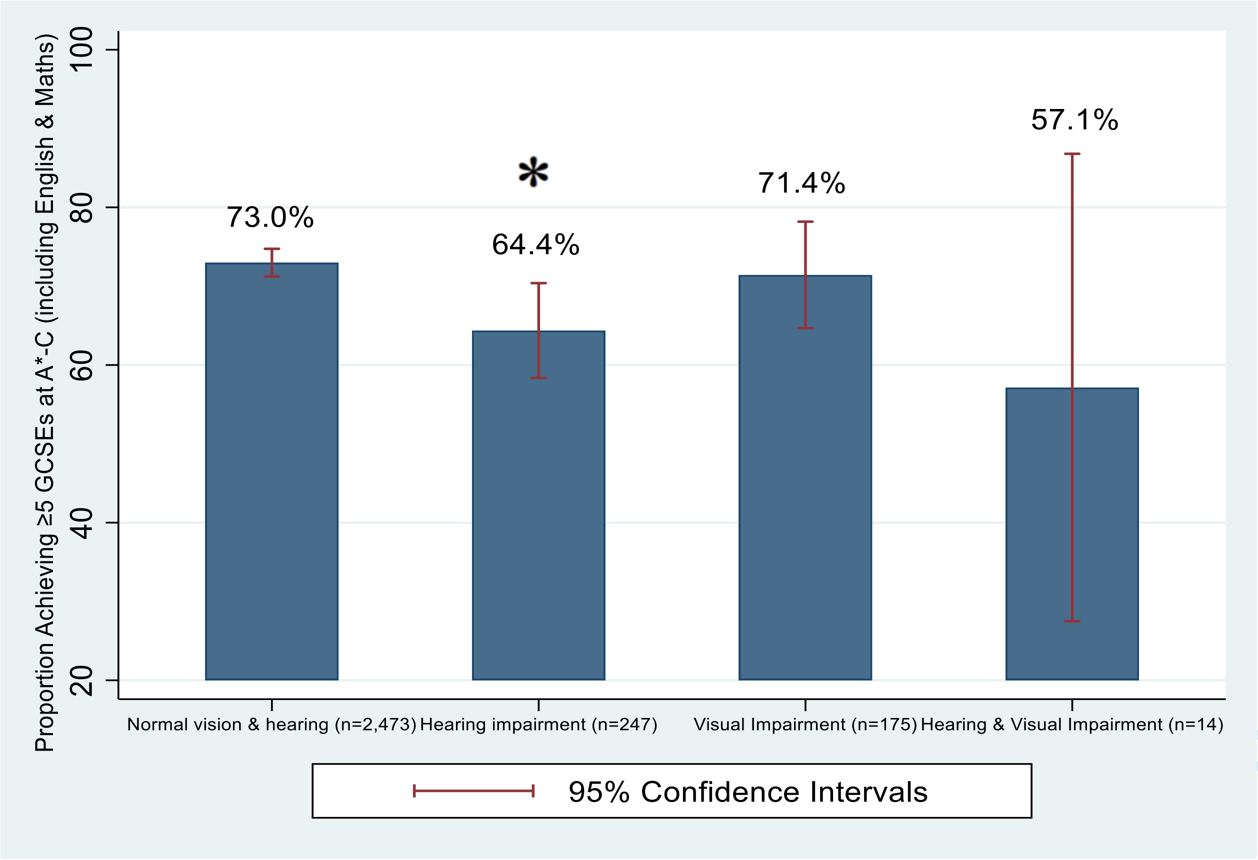
Proportion Achieving ≥5 GCSEs at A*-C (including English & Maths)
100
80
60
40
20
73.0%
*
64.4%
71.4%
57.1%
Normal vision & hearing (n=2,473)
Hearing impairment (n=247)
Visual Impairment (n=175)
Hearing & Visual Impairment (n=14)
95% Confidence Intervals

w Only

Supplementary Material

Appendix A Hearing and vision assessment

The hearing assessment was carried out by audiologists and specially trained staff, and performed as per the British Society of Audiologists standards(13). Air conduction hearing thresholds were measured bilaterally at 500 Hz, 1kHz, 2kHz, 4kHz and 8kHz, and bone conduction thresholds were measured at 4 kHz for all children and at 1kHz if time permitted. Tympanometry was used to measure middle ear function bilaterally. Tympanograms were categorised according to the Jerger's modified Fieullau-Nikolajsen method(14), with type B tympanograms indicating the presence of otitis media with effusion (OME, 'glue ear').

The visual tests were undertaken by trained orthoptists and overseen by an ophthalmologist. Visual acuity was measured monocularly using the LogMAR test and was taken as the best of presenting (with glasses if worn) and presenting-plus-pinhole. Ocular misalignment was assessed using a simultaneous prism cover test and alternate prism cover test, and strabismus was classified according to the direction (convergent, divergent, vertical or mixed) and whether it was manifest (present in normal viewing with both eyes open), or latent (present only when the eyes were dissociated and/or one covered). Additional information regarding previous visual difficulties was obtained via a questionnaire.

Appendix B Mediating factors

Intelligence quotient (IQ), attention, social cognition and behaviour were selected as potential mediating factors. Information on these domains was collected during a research clinic to which all ALSPAC participants were invited to attend at age 8. IQ was tested using a shortened form of the Wechsler Intelligence Scale for Children, 3rd edition (WISC-III)(31); this comprised five verbal subtests and five performance subtests from which a total age-scaled score was derived. Selective attention was assessed using the Sky Search Test, taken from the Test of Everyday Attention for children(32). The Diagnostic Analysis of Non-Verbal Accuracy (DANVA) faces subtest was used to test social cognition; we used a binary measure comprising the proportion of children with >=7 errors on the facial expressions recognition task(33). Finally, behaviour was assessed using the Strength and Difficulties Questionnaire (SDQ), completed by parents when children were 9 years of age(34). This comprises questions relating to hyperactivity, emotional symptoms, conduct and peer problems.

Appendix C Multiple imputation for missing data

To minimise the potential bias introduced by missing data, we performed multiple imputation using the mi impute command in Stata v.15.0. The exposure, confounding and mediating factors and outcome variables included in the final model were imputed in the sample of 8,205 that attended the research clinic at 7 years and were eligible for inclusion in the study sample. **In addition, we identified factors which predicted missingness using logistic regression analyses and imputed these variables (maternal smoking during pregnancy, male gender and maternal age <25 years). All of these variables were included in the final model, thus the assumption of "missing at random" is supported.**

Confidential: For Review Only

Table ST1 summarises the variables and proportion of missing values and compares observed distributions to their distributions after the multivariate imputation for used to impute missing values.

Variable	Number missing (%)	Observed data	Complete data
		% for categorical/binary variables or mean (SD) for continuous variables	
**Hearing impairment** (OME and/or conductive hearing loss)	802 (9.8%)	10.3	10.4
**Visual impairment** (Amblyopia and/or strabismus and/or reduced acuity)	1005 (12.2%)	6.9	7.0
**Maternal age <25 years**	451 (5.5%)	21.6	21.8
**Admission to SCBU**	821 (10.0%)	6.7	6.9
**Ethnicity – white**	860 (10.5%)	96.0	95.9
**Maternal smoking during pregnancy**	1410 (17.2%)	20.0	20.3
**Achievement of ≥ Level 4 at KS2 English**	49 (0.6%)	75.2	75.2
**Achievement of ≥ Level 4 at KS2 Maths**	1050 (12.8%)	82.9	81.9
**Achievement of ≥ Level 4 at KS2 Science**	47 (0.6%)	82.9	83.0
**Achievement of ≥ 5 GCSEs at A*- C** (including Maths & English)	1256 (15.3%)	59.8	56.3
**DANVA** (≥7 mistakes)	2038 (24.8%)	22.3	23.3
**Family Adversity Index (1 – 12)**	643 (7.8%)	1.75 (1.89)	1.75 (1.89)
**Wechsler Intelligence Scale (WISC-III)**	1547 (18.9%)	104.7 (16.3)	104.5 (16.4)
**Strengths & Difficulties Questionnaire (SDQ)**	1703 (20.8%)	6.75 (4.84)	6.77 (4.96)
**Sky Search Test**	1710 (20.8%)	5.20 (1.9)	5.21 (2.0)
**Index of Multiple Deprivations**	1968 (24.0%)		
**1**		33.1	32.2
**2**		19.6	19.3
**3**		18.9	18.9
**4**		15.3	15.8
**5**		13.1	13.8
**Parity**	716 (8.7%)		
**0**		46.1	46.3
**1**		36.0	35.8
**>=2**		17.9	17.9

Table ST1: Proportion of data missing for the variables imputed, and distribution of values in the observed data and after imputation

Confidential: For Review Only

Characteristic	Attended clinic at 7 years (N = 8,283)		Did not attend clinic at 7 years (N = 6,402)		P-value
	N	Percentage of complete data (%)	N	Percentage of complete data (%)	
**Sex**					
Male	4,201	50.7	3,335	52.1	0.09
Female	4,082	49.3	3,066	47.9	
**Low birthweight (<2,500g)**					
Yes	377	4.9	370	6.1	0.00
Missing	549	-	343	-	
**Premature (gestation <37 weeks)**					
Yes	451	5.8	394	6.4	0.11
Missing	455	-	257	-	
**Admission to SCBU**					
Yes	507	6.8	355	7.5	0.17
Missing	830	-	1,644	-	
**Ethnicity**					
White	7,109	95.9	4,359	93.4	0.00
Non-white	301	4.1	308	6.6	
Missing	873	-	1,734	-	
**Parity**					
0	3,477	42.0	2,293	35.8	
1	2,720	32.8	1,819	28.4	0.00
≥2	1,362	16.4	1,253	16.4	
Missing	724	-	1,760	-	
**Maternal age**					
Under 25 years	1,687	21.6	2,602	42.4	0.00
Missing	455	15.6	257	4.0	
**Breastfeeding**					
Yes	3,346	40.4	1,787	27.9	
Missing	4,937	59.6	4,614	72.1	0.00
**Maternal smoking during pregnancy**					
Yes	1,371	20.0	1,529	36.0	0.00
Missing	1,429	-	2,157	-	
**Family Adversity Index at 2-4 years**					
<3	5,646	74.0	3,096	63.3	0.00
≥3	1,987	26.0	1,794	36.7	
Missing	651	-	1,511	-	
**Index of Multiple Deprivations at 7 years**					
1	2,079	33.0	318	23.2	0.00
≥2	4,213	67.0	1,054	76.8	
Missing	1,991	-	5,029	-	
**KS2 ≥ level 4**					
**English**					
Yes	6,172	75.0	3,616	56.9	0.00
Missing	995	-	1,653	-	
**Maths**					
Yes	5,962	82.6	3,536	69.1	0.00
Missing	1,050	-	1,212	-	
**Science**					
Yes	6,810	82.7	4,346	31.7	0.00
Missing	992	-	1,178	-	
**GCSEs ≥5 grade A*-C including Maths & English**					
Yes	4,177	59.5	1,875	37.2	0.00
**SEN at KS2**					
Yes (School action, Action plus or SEN)	948	13.7	1,162	22.8	
Missing	1,379	-	1,307	-	
**SEN at KS4**					
Yes (School action, Action plus or SEN)	733	11.3	859	18.2	
Missing	1,813	-	1,689	-	

Table ST2: Demographic characteristics of children that attended the ALSPAC research clinic at 7 years compared to those that did not attend. The student's T-test was used to calculate p-values.

Confidential: For Review Only

Characteristic	Normal Vision & Hearing (n= 2,473)		Hearing Impairment alone (n=247)			Visual Impairment alone (n=175)			Co-occurring hearing & visual Impairment (n=14)		
	N	Percentage (%)	N	Percentage (%)	P-value	N	Percentage (%)	P-value	N	Percentage (%)	P-value
**Sex**<br>Male<br>Female	1,218<br>1,255	49.3<br>50.7	125<br>122	50.6<br>49.4	0.68	79<br>96	45.1<br>54.9	0.29	10<br><5	71.4<br>28.6	0.10
**Low birthweight (<2,500g)**	89	3.6	10	4.1	0.72	11	6.3	0.07	<5	7.1	0.48
**Premature (gestation <37 weeks)**	121	4.9	16	6.5	0.28	11	6.3	0.41	<5	14.3	0.11
**Admission to SCBU**	134	5.4	23	9.3	0.01	11	6.3	0.62	<5	0	0.37
**Ethnicity**<br>White<br>Non-white	2,398<br>75	97.0<br>3.0	241<br>6	97.6<br>2.4	0.60	172<br><5	98.3<br>1.7	0.32	14<br><5	100<br>0	0.51
**Parity**<br>0<br>1<br>≥2	1,122<br>920<br>431	45.4<br>37.2<br>17.4	121<br>79<br>47	49.0<br>32.0<br>19.0	0.28<br>0.11<br>0.53	77<br>78<br>20	44.0<br>44.6<br>11.4	0.73<br>0.05<br>0.04	6<br>6<br><5	42.9<br>42.9<br>14.3	0.85<br>0.66<br>0.76
**Maternal age <25 years**	452	18.3	48	19.4	0.65	39	22.3	0.19	<5	14.3	0.70
**Breastfed**	1,103	44.6	106	42.9	0.61	85	48.6	0.31	<5	28.6	0.23
**Maternal smoking during pregnancy**	445	18.0	39	15.8	0.39	43	24.6	0.03	5	35.7	0.09
**Family Adversity Index at 2-4 years**<br><3<br>≥3	2,089<br>384	84.5<br>15.5	205<br>42	83.0<br>17.0	0.54	139<br>36	79.4<br>20.6	0.08	11<br><5	78.6<br>21.4	0.54
**Index of Multiple Deprivations at 7 years**<br>1<br>≥2	822<br>1,652	33.2<br>66.8	72<br>175	29.2<br>70.9	0.19	54<br>121	30.9<br>69.1	0.52	5<br>9	35.7<br>64.2	0.84

Table ST3: Demographic characteristics of children according to their hearing and vision status. NB. "<5" may include zero. The student's T-test was used to calculate p-values.

	KS2 English		KS2 Maths		KS2 Science	
	N	Percentage (95% CI)	N	Percentage (95% CI)	N	Percentage (95% CI)
**Normal vision & hearing N=2,473**	2,306	93.3<br>(92.3 – 94.2)	2,429	98.2<br>(97.7 – 98.7)	2,421	97.9<br>(97.3 – 98.5)
**Hearing impairment N= 247**	226	91.5<br>(88.0 – 95.0)	239	96.8<br>(94.5 – 99.0)	241	97.6<br>(95.6 – 99.5)
**Visual impairment N= 175**	149	85.1<br>(79.8 – 90.5	161	92.0<br>(87.9 – 96.1)	165	94.3<br>(90.8 – 97.8)
**Co-occurring hearing & visual impairment N=14**	10	71.4<br>(44.4 – 98.5)	12	85.7<br>(64.8 - >100)	13	92.9<br>(77.4 - >100)

Table ST4: Proportions of children achieving level 4 or above at Key Stage 2 English, Maths and Science by hearing and vision status

	Normal hearing & vision N = 2480	Hearing impairment N= 247	Visual impairment N= 175	Co-occurring hearing & visual impairment N=14	P-value
**SEN support at KS2**<br>Number<br>Percent (%)<br>(95% CI)	153<br>6.2<br>(5.2 – 7.7)	21<br>8.5<br>(5.4 – 12.3)	20<br>11.4<br>(7.0 – 16.3)	<5<br>14.3<br>(<0 – 35.3)	0.02
**SEN support at KS4**<br>Number<br>Percent (%)<br>(95% CI)	147<br>5.94<br>(5.0 – 6.9)	17<br>9.7%<br>(5.4 – 12.3)	21<br>11.05<br>(6.6 – 15.6)	<5<br>28.6<br>(1.5 – 55.6)	0.01

Table ST5: Proportions of children receiving SEN support at KS2 & KS4 by hearing and vision status. The Pearson's chi squared test was used to calculate p-values.

Table ST6: Multivariate regression models using the imputed dataset (N=8,205)

**KS2 Maths: Achievement of ≥level 4**												
	**Univariate logistic regression**		**Model 1** : Male, age at testing, SEN at KS2		**Model 2:** Model 1 & maternal age <25, parity & smoking during pregnancy		**Model 3:** Model 2 & FAI & IMD		**Model 4:** Model 3 & IQ		**Model 5:** Model 4 & behaviour, social perceptions & attention	
	**OR**	**95% CI**	**OR**	**95% CI**	**OR**	**95% CI**	**OR**	**95% CI**	**OR**	**95% CI**	**OR**	**95% CI**
**Hearing impairment** (n=852)	0.83	0.68 – 1.01	0.90	0.71 – 1.15	0.90	0.71 – 1.15	0.90	0.71 – 1.15	0.91	0.72 – 1.16	0.91	0.71 – 1.15
**Visual impairment** (n=573)	0.66	0.53 – 0.83	0.81	0.61 – 1.07	0.80	0.60 – 1.06	0.81	0.61 – 1.07	0.81	0.61 – 1.08	0.82	0.61 – 1.08
**Co-occurring hearing & visual impairment** (n=45)	0.33	0.17 – 0.63	0.47	0.22 – 1.01	0.46	0.21 – 1.01	0.46	0.21 – 1.01	0.46	0.21 – 1.00	0.46	0.21 – 1.00

demonstrating the likelihood children achieved ≥level 4 at KS2 Maths according to hearing and visual status and after adjustment for confounding variables (models 1-3) and mediating factors (models 4-5). OR=Odds Ratio

Confidential: For Review Only

KS2 Science: Achievement of ≥level 4												
	**Univariate logistic regression**		**Model 1** : Male, age at testing, SEN at KS2		**Model 2:** Model 1 & maternal age <25, parity & smoking during pregnancy		**Model 3:** Model 2 & FAI & IMD		**Model 4:** Model 3 & IQ		**Model 5:** Model 4 & behaviour, social perceptions & attention	
	**OR**	**95% CI**	**OR**	**95% CI**	**OR**	**95% CI**	**OR**	**95% CI**	**OR**	**95% CI**	**OR**	**95% CI**
**Hearing impairment** (n=852)	0.89	0.73 – 1.08	0.97	0.77 – 1.22	0.97	0.77 – 1.22	0.97	0.77 – 1.22	0.99	0.78 – 1.24	0.99	0.78 – 1.24
**Visual impairment** (n=573)	0.69	0.54 – 0.87	0.83	0.64 – 1.08	0.84	0.65 – 1.09	0.84	0.65 – 1.10	0.86	0.66 – 1.12	0.87	0.66 – 1.13
**Co-occurring hearing & visual impairment** (n=45)	0.33	0.18 – 0.61	0.46	0.22 – 0.95	0.47	0.23 – 0.97	0.47	0.23 – 0.97	0.48	0.23 – 0.99	0.48	0.23 – 0.99

Table ST7: Multivariate regression models using the imputed dataset (N=8,205) demonstrating the likelihood children achieved ≥level 4 at KS2 Science according to hearing and visual status and after adjustment for confounding variables (models 1-3) and mediating factors (models 4-5). OR=Odds Ratio

Confidential: For Review Only

KS2 English: Achievement of ≥level 4								
	**Univariate logistic regression**		**Model 1** : Male, age at testing, SEN at KS2		**Model 2:** Model 1 & maternal age <25, parity & smoking during pregnancy		**Model 3**: Model 2 & FAI & IMD	
	**OR**	**95% CI**	**OR**	**95% CI**	**OR**	**95% CI**	**OR**	**95% CI**
**Hearing impairment** (n=261)	0.74	0.48 – 1.15	0.81	0.50 – 1.32	0.80	0.49 – 1.31	0.76	0.47 – 1.24
**Visual impairment** (n=189)	0.39	0.30 – 0.60	0.42	0.26 – 0.67	0.42	0.26 – 0.68	0.42	0.26 – 0.68
**Co-occurring hearing & visual impairment** (n=14)	0.20	0.06 – 0.64	0.21	0.06 – 0.81	0.21	0.05 – 0.84	0.21	0.05 – 0.83
**KS2 Maths: Achievement of ≥level 4**								
	**Univariate logistic regression**		**Model 1** : Male, age at testing, SEN at KS2		**Model 2:** Model 1 & maternal age <25, parity & smoking during pregnancy		**Model 3:** Model 2 & FAI & IMD	
	**OR**	**95% CI**	**OR**	**95% CI**	**OR**	**95% CI**	**OR**	**95% CI**
**Hearing impairment** (n=261)	0.56	0.28 – 1.11	0.64	0.30 – 1.33	0.66	0.31 – 1.40	0.58	0.27 – 1.23
**Visual impairment** (n=189)	0.21	0.12 – 0.38	0.24	0.12 – 0.46	0.21	0.11 – 0.41	0.20	0.10 – 0.41
**Co-occurring hearing & visual impairment** (n=14)	0.14	0.03 – 0.64	0.15	0.02 – 0.91	0.13	0.02 – 0.87	0.10	0.02 – 0.57
**KS2 Science: Achievement of ≥level 4**								
	**Univariate logistic regression**		**Model 1** : Male, age at testing, SEN at KS2		**Model 2:** Model 1 & maternal age <25, parity & smoking during pregnancy		**Model 3:** Model 2 & FAI & IMD	
	**OR**	**95% CI**	**OR**	**95% CI**	**OR**	**95% CI**	**OR**	**95% CI**
**Hearing impairment** (n=261)	0.87	0.39 – 1.92	1.00	0.44 – 2.28	1.00	0.43 – 2.31	0.89	0.38 – 2.08
**Visual impairment** (n=189)	0.35	0.18 – 0.68	0.46	0.23 – 0.94	0.49	0.23 – 1.03	0.53	0.24 – 1.16
**Co-occurring hearing & visual impairment** (n=14)	0.31	0.04 – 2.42	0.38	0.04 – 3.57	0.49	0.05 – 5.05	0.35	0.04 – 3.21

Table ST8-ST10: Multivariate regression models using complete cases (N=2,909) demonstrating the likelihood children achieved ≥level 4 at KS2 English, Maths & Science according to hearing and visual status and after adjustment for confounding variables (models 1-3). OR=Odds Ratio

Confidential: For Review Only

KS4: Achievement of ≥5 GCSEs at A*-C, including Maths & English										
	**Univariate logistic regression**		**Model 1 :** Male, SCBU admission, SEN at KS4		**Model 2:** Model 1 & maternal age <25, parity & smoking during pregnancy		**Model 3:** Model 2 & FAI & IMD		**Model 4:** Model 3 & KS2 English, Maths & Science performance	
	**OR**	**95% CI**	**OR**	**95% CI**	**OR**	**95% CI**	**OR**	**95% CI**	**OR**	**95% CI**
**Hearing impairment** (n=261)	0.66	0.51 – 0.86	0.69	0.52 – 0.91	0.66	0.50 – 0.88	0.64	0.48 – 0.86	0.64	0.47 – 0.87
**Visual impairment** (n=189)	0.91	0.66 – 1.26	1.00	0.71 – 1.41	1.08	0.76 – 1.53	1.12	0.77 – 1.59	1.47	0.98 – 2.21
**Co-occurring hearing & visual impairment** (n=14)	0.51	0.18 – 1.49	0.79	0.25 – 2.53	0.82	0.24 – 2.79	0.77	0.22 – 2.64	1.18	0.29 – 4.74

Table ST11: Multivariate regression models using complete cases (N=2,909) demonstrating the likelihood children achieved ≥level 4 at KS2 English, Maths & Science and ≥5 GCSEs at A-C grade (including Maths & English), according to hearing and visual status and after adjustment for. OR=Odds Ratio*

Confidential: For Review Only

BMJ Paediatrics Open

THE IMPACT OF CO-OCCURING HEARING AND VISUAL DIFFICULTIES IN CHILDHOOD ON EDUCATIONAL OUTCOMES: A LONGITUDINAL COHORT STUDY

Journal:	*BMJ Paediatrics Open*
Manuscript ID	bmjpo-2018-000389.R2
Article Type:	Original article
Date Submitted by the Author:	30-Jan-2019
Complete List of Authors:	Hill, Matilda; University of Bristol Centre for Child and Adolescent Health<br>Hall, Amanda; Aston University School of Life and Health Sciences<br>Williams, Cathy; University of Bristol Centre for Child and Adolescent Health<br>Emond, Alan; University of Bristol, Centre for Child and Adolescent Health
Keywords:	Comm Child Health, Ophthalmology, Audiology, Epidemiology, Paediatric Practice

SCHOLARONE™
Manuscripts

THE IMPACT OF CO-OCCURING HEARING AND VISUAL DIFFICULTIES IN CHILDHOOD ON EDUCATIONAL OUTCOMES: A LONGITUDINAL COHORT STUDY

Matilda Hill[1], Amanda Hall[2], Cathy Williams[1], Alan Emond[1]

matildahill84@gmail.com, a.hall@aston.ac.uk, cathy.williams@bristol.ac.uk, alan.emond@bristol.ac.uk

Correspondence to: Dr Matilda Hill, Centre for Academic Child Health, Bristol Medical School, University of Bristol BS8 1NU

Confidential: For Review Only

[1] Centre for Academic Child Health, Bristol Medical School, University of Bristol BS8 1NU
[2] Life and Health Sciences, Aston University, Birmingham B4 7ET

What is known about the subject

- Mild hearing and visual difficulties are common in childhood.
- The most common cause of hearing difficulties in childhood is chronic otitis media with effusion (OME), which may be associated with lower academic performance.
- The impact of common co-occurring hearing and visual difficulties on educational outcomes is not known.

What this study adds

- Children with co-occurring mild hearing and visual difficulties have poorer educational outcomes than children with isolated hearing or visual difficulties.
- Mild visual difficulties alone do not negatively impact on academic performance at school.

Confidential: For Review Only

Abstract

Background: Mild hearing and visual difficulties are common in childhood, and both may have implications for educational achievement. However, the impact of co-occurring common hearing and visual difficulties in childhood is not known.

Objective: To determine the prevalence and impact of co-occurring common hearing and visual difficulties of childhood on educational outcomes in primary and secondary school.

Methods: The sample was drawn from the Avon Longitudinal Study of Parents and Children (ALSPAC), a longitudinal birth cohort study in England. The exposures were hearing and visual difficulties at age 7 (defined as conductive hearing loss or OME, and amblyopia, strabismus or reduced visual acuity, respectively). The outcomes measured were achievement of level 4 or above at Key Stage 2 (KS2) in English, Maths and Science, respectively, at age 11, and attainment of 5 or more GCSEs at grades A*-C at age 16. Multiple logistic regression models assessed the relationship between hearing and visual difficulties and educational outcomes, adjusting for potential confounding factors.

Results: 2,909 children were included in the study; 261 had hearing difficulties, 189 had visual difficulties and 14 children had co-occurring hearing and visual difficulties. Children with co-occurring hearing and visual difficulties were less likely to achieve the national target at KS2 compared to children with normal hearing and vision, even after adjustment for confounding factors (OR 0.30, CI 0.15-0.61 for KS2 English). Differences in IQ, behaviour, attention and social cognition did not account for this relationship. The impact of co-occurring hearing and visual difficulties on GCSE results was explained largely by poor performance at KS2.

Conclusions: Co-occurring hearing and visual difficulties in childhood have an enduring negative impact on educational outcomes. Identification of affected children and early intervention in primary school is essential.

Confidential: For Review Only

Confidential: For Review Only

Introduction

Hearing and visual difficulties are prevalent amongst children in the UK. The majority of affected children have mild, temporary difficulties, which may not be formally diagnosed and for which they do not receive additional educational support. However, there is evidence that even mild deficits in hearing or vision can have implications for learning and development throughout childhood(1-5).

The most common cause of chronic hearing loss in childhood is persistent otitis media with effusion (OME, 'glue ear'). There is ongoing controversy regarding the impact of OME on language development and cognition. Whilst several large studies have found no association between OME and language or academic attainment(6, 7), few of these studies have considered concurrent hearing loss or chronicity of OME. Conversely, a number of studies have demonstrated an association between persistent OME-related hearing loss, cognitive development, reading ability and behavioural problems (2-4, 8).

The impact of common visual difficulties on academic attainment in childhood is not well characterised(9). Decreased visual acuity in childhood is associated with reduced literacy(1), but the consequences of more common visual difficulties are not clear. Amblyopia and strabismus are two of the most common visual difficulties encountered in childhood(10), which often occur in the context of conditions which may in themselves affect development and educational achievement such as prematurity and low birthweight(11, 12), thus rigorous adjustment for confounding factors is required to establish the functional impact of these visual difficulties in of themselves.

The impact of co-occurring common hearing and visual difficulties on educational outcomes in childhood has not previously been studied. However, it is well-established that integration of auditory and visual information is essential for many cognitive processes, in particular speech perception, and key for the development of language and communication skills(13, 14). A deficit in one of these

Confidential: For Review Only

domains in childhood may therefore hinder developing literacy and render an individual vulnerable to the cumulative effect of an additional sensory deficit.

The aim of this study is to investigate the prevalence and impact of co-occurring common hearing and visual difficulties in childhood on educational outcomes at primary school and secondary school using a large population cohort. We hypothesise that children with co-occurring mild hearing and visual difficulties are more likely to have poorer language and communication skills, and thus lower levels of academic attainment, relative to those with a single sensory deficit or children with normal hearing and vision.

Methods

Study Participants

The Avon Longitudinal Study of Parents and Children (ALSPAC) is a longitudinal population-based birth cohort study. All pregnant women resident in Avon, UK, with expected dates of delivery between 1st April 1991 and 31st December 1992 were eligible for participation, resulting in enrolment of over 14,000 live births(15, 16). Ethical approval for the study was obtained from the ALSPAC Ethics and Law Committee and the Local Research Ethics Committees.
Please note that the study website contains details of all the data that is available through a fully searchable data dictionary and variable search tool: http://www.bristol.ac.uk/alspac/researchers/our-data/

Patient and Public Involvement

ALSPAC participants advise on ALSPAC studies through the original cohort advisory panel (OCAP) and contribute to the ALSPAC ethics and law committee. ALSPAC participants were not directly involved in the design of this study.

Hearing and vision assessments

Confidential: For Review Only

All participating children in ALSPAC were invited to attend a research clinic at 7 years of age. 59.3% (8,299 children) attended during the period September 1998 - September 2000, of whom 98.9% (8,205 children) were eligible for inclusion (see Figure 1). The clinics included a comprehensive assessment of vision and hearing, details of which are included in Appendix A of the Supplementary Material.

Definition of Hearing Difficulties

Hearing difficulties were defined as the presence of mild-moderate conductive hearing loss and/or OME in either ear, characterised by air conduction greater than 20 dB and less than 70 dB averaged across 500 Hz, 1kHz, 2kHz and 4kHz (based on The British Society of Audiology definitions(17)), or the presence of a type B tympanogram, respectively. The prevalence of OME decreases with age after the first 2 years of life(18); children with evidence of OME at age 7 are likely to have persistent OME which is associated with conductive hearing loss in over 70% of cases(19, 20). However, hearing loss may be fluctuant and thus not captured by a single clinic assessment, hence the utilisation of both air conduction tests and tympanometry to identify children with hearing difficulties.

A total of 5 children with sensorineural hearing loss, defined by bone conduction greater than 30 dB at either 1 kHz or 4 kHz, were excluded from this analysis.

Definition of Visual Difficulties

Children with "clinically significant" strabismus, amblyopia or mild-moderate reduced acuity (based on the WHO ICD 11 definition(21)) were defined as having visual difficulties. "Clinically significant" strabismus comprised all children with manifest strabismus, or previously defined large latent deviations (>=10 pd if convergent & >=15pd if divergent)(10). Amblyopia was defined as a history of patching treatment and/or an interocular difference in acuity of >0.2logMAR units where the worst-seeing eye had an acuity of >0.3logMAR. Reduced acuity was defined by reduced distance acuity of the better-seeing eye >=0.3 logMAR. Acuity was assessed with glasses if worn ('habitual' state), and in the 'habitual state plus pinhole', as a proxy for full refractive correction.

Confidential: For Review Only

Refractive errors were not included in our definition of visual difficulties as these are potentially correctable with glasses and have already been studied extensively in the context of educational achievement, with myopia being linked with higher educational achievement(22, 23).

19 children with known ocular pathology or severe visual impairments (>1.0logMAR) were excluded. Triplets, quadruplets and children with Down's Syndrome or Cerebral Palsy were also excluded from this analysis.

Educational Outcomes

Educational outcomes at primary school were assessed using Standardised Assessment Test (SAT) results, obtained from the National Pupil Database (NPD)(24). Key Stage 2 (KS2) tests are undertaken during the final year of primary school (year 6) at age 10-11. The national expected standard is achievement of National Curriculum level 4 or above, and we therefore used achievement of ≥level 4 at KS2 in English, Maths and Science as our three educational outcomes in primary school; performance in each subject was analysed separately.

Educational outcomes in secondary school were assessed using General Certificate of Secondary Education (GCSE) results at Key Stage 4 (KS4). GCSEs are taken at the end of compulsory schooling (year 11) at age 16, and are graded A*- G. Achievement of 5 or more GCSEs (including Maths and English) at grades A*- C is the national benchmark measure of achievement, and we used this as our single outcome at secondary school. We adjusted outcomes for KS2 attainment, as performance at KS2 is well known to predict performance at GCSEs(25).

Information on children receiving Special Educational Needs (SEN) support was provided by the Pupil Level Annual School Census (PLASC). SEN status was obtained for each child at KS2 and KS4, with information on the level of individual educational support being provided. We

Confidential: For Review Only

dichotomised these data into children receiving no special provision and those receiving some level of support.

NPD and PLASC data were not available for children attending independent schools, or schools outside of England, and these children were excluded from this analysis.

Confounding and Mediating Factors

Gender, prematurity, low birthweight, admission to a Special Care Baby Unit (SCBU), maternal age, parity, smoking during pregnancy, duration of breastfeeding and socioeconomic status were selected as potential confounding factors, based on their previously identified associations with hearing or visual difficulties in childhood and well-established links with academic performance(10-12, 26-29). The ALSPAC Family Adversity Index (FAI), derived from a questionnaire about socioeconomic status completed by mothers at 2-4 years, was used as a measure of family adversity. The Indices of Multiple Deprivations (IMD) at age 7, a census-derived score of relative deprivation of a neighbourhood was used as a measure of social deprivation.

Intelligence quotient (IQ), attention, social cognition and behaviour were selected as potential mediating factors, as they may be influenced by visual and hearing difficulties and can impact on educational achievement(1, 3, 4, 8). Reading ability was not included due to high rates of missing data. These domains were tested during a research clinic to which all participating children were invited and 7,488 children (53.5%) attended at 8 years of age. Further information on these assessments is provided in Appendix B of the Supplementary Material.

Analyses

Binary univariate logistic regressions were used to calculate odds ratios (OR) and 95% confidence intervals (95% CI) to assess the relationship between hearing and visual difficulties and educational outcomes. The analyses were repeated, controlling for all potential confounding factors significant at the 5% level in the univariate analyses and all potential mediating factors which fulfilled the Baron

Confidential: For Review Only

and Kenny mediation model steps 1-3(30). These multiple logistic regression models sequentially adjusted for individual factors, maternal factors, wider socioeconomic factors, earlier educational performance, IQ and additional mediating factors. Further information is provided in Tables 2 & 3.

Multiple imputation using chained equations (ICE) was used to impute missing data for all variables included in the final logistic regression models, including the outcome, and variables that predicted missingness. This technique helps to minimise attrition bias and improve precision of estimates(31). We imputed data for all 8,205 children who attended the research clinic at 7 years and did not meet the exclusion criteria. Twenty imputations were performed. All analyses were carried out using STATA version 15.0 (StataCorp 2017). Further information regarding the multiple imputation is provided in Appendix C of the Supplementary Material.

Results

Sample Characteristics

Of the 8,205 children that attended the research clinic at 7 years and were eligible for inclusion, 2,909 (33.5%) had complete data for hearing and vision, educational outcomes and potential confounding variables (see Figure 1).

Children attending the research clinic were more likely to be come from families of higher socioeconomic class and achieve higher levels of academic attainment at both KS2 and KS4 than children that did not attend the clinic (see supplementary material, Table ST2).

Prevalence and Causes of Hearing & Visual Difficulties

Of the 2,909 children with complete data, 261 (9.0%) had hearing difficulties, 189 (6.5%) had visual difficulties and 14 (0.5%) had both hearing and visual difficulties. Types of hearing and visual difficulties are demonstrated in Table 1.

Confidential: For Review Only

Conductive hearing loss was identified in 159 children; 145 of these children (91%) had mild hearing loss (air conduction of 21.25 – 40 dB, mean 27.68dB), and 14 children had moderate hearing loss (air conduction 41.25dB – 66.25dB, mean 46.25dB). The majority of children with conductive hearing loss had evidence of concurrent OME.

The overall prevalence of OME in either or both ears was 6.7%. A higher proportion of children undergoing assessment in winter months had OME compared to those attending clinic in the summer months (8.0% vs 5.6%), but this association was weak (p=0.09).

**Types of Hearing and Visual Difficulties**	**Proportion of children with either hearing or visual difficulties (%)**
**Hearing Difficulties**	**261 (100%)**
Otitis Media with Effusion	102 (39.1%)
Otitis Media with Effusion & Conductive Hearing Loss	94 (36.0%)
Conductive Hearing Loss	65 (24.9%)
**Visual Difficulties**	**189 (100%)**
Amblyopia	79 (41.8%)
Strabismus	56 (29.6%)
Strabismus & Amblyopia	47 (24.9%)

Confidential: For Review Only

Reduced Acuity with or without Amblyopia and/or Strabismus	7 (3.7%)

Table 1: Types of hearing and visual difficulties

Amblyopia was the most common cause of visual difficulties, affecting 4.4% of all children. There was considerable overlap between amblyopia and strabismus; of the 128 children with amblyopia, 47 (36.7%) also had strabismus.

Only 7 children had reduced habitual acuity in the best-seeing eye; 4 children had mild reduced visual acuity (0.3logMAR – 0.44logMAR), and 3 children had moderate reduced acuity (0.50logMAR – 0.7logMAR), with a mean acuity of 0.45logMAR. These reduced acuities most likely represent uncorrected refractive errors, given that acuity was not assessed with full refractive correction and the exclusion of children with ocular pathology.

Children with hearing and visual difficulties had similar characteristics to those with normal hearing and vision (see supplementary material, Table ST3). However, children with visual difficulties were more likely to have mothers who smoked during pregnancy (24.6% vs 18.0%, p=0.03) and children with hearing difficulties were more likely to have been admitted to SCBU in infancy (9.3% vs 5.4%, p=0.01).

Educational Outcomes and SEN status in Primary School

The proportion of children in this study achieving level 4 or above at KS2 exceeded the national average; 92.5% and 97.7% of children achieved level 4 or above in KS2 English and Maths tests respectively, compared to 75% and 73% of children nationally(32). The proportions of children achieving these targets by hearing and visual status are shown in supplementary material, Table ST4.

Confidential: For Review Only

Children with either hearing or visual difficulties were less likely to achieve level 4 or above at KS2 English tests relative to children with normal hearing and vision (see Table 2 for the imputed dataset analysis; see supplementary material, Table ST8-ST10 for the complete cases analysis). However, these relationships were attenuated by adjustment for confounding factors, and further attenuated after adjustment for IQ, suggesting that some of the association is mediated through IQ. In contrast, children with co-occurring hearing and visual difficulties remained less likely to achieve this target after controlling for confounding factors (OR 0.30, CI 0.15-0.61). This relationship was not significantly altered by adjustment for IQ or other potential mediating factors.

**KS2 English: Achievement of ≥level 4**												
	**Univariate logistic regression**		**Model 1** : Male, age at testing, SEN at KS2		**Model 2:** Model 1 & maternal age <25, parity & smoking during pregnancy		**Model 3:** Model 2 & FAI & IMD		**Model 4:** Model 3 & IQ		**Model 5:** Model 4 & behaviour, social perceptions & attention	
	**OR**	**95% CI**	**OR**	**95% CI**	**OR**	**95% CI**	**OR**	**95% CI**	**OR**	**95% CI**	**OR**	**95% CI**
**Hearing difficulties** (n=852)	0.78	0.66 – 0.92	0.80	0.66 – 0.98	0.81	0.66 – 0.99	0.81	0.66 – 0.99	0.87	0.71 – 1.06	0.88	0.72 – 1.08

Confidential: For Review Only

**Visual difficulties** (n=573)	0.66	0.64 – 0.80	0.79	0.62 – 0.99	0.81	0.64 – 1.02	0.82	0.64 – 1.03	0.87	0.68 – 1.11	0.90	0.71 – 1.14
**Co-occurring hearing & visual difficulties** (n=45)	0.25	0.14 – 0.45	0.30	0.15 – 0.61	0.30	0.15 – 0.61	0.30	0.15 – 0.61	0.36	0.17 – 0.74	0.38	0.18 – 0.78

Table 2: Multiple regression models using the imputed dataset (N=8,205) demonstrating the likelihood children achieved ≥level 4 at KS2 English according to hearing and visual status and after adjustment for confounding variables (models 1-3) and mediating factors (models 4-5). OR=Odds Ratio

The relationship is similar for performance in KS2 Maths and KS2 Science tests, albeit hearing difficulties appear to have less of an impact (see supplementary material, Table ST6 and ST7).

The proportion of children with visual difficulties receiving SEN support was higher compared to children with normal hearing and vision (11.4% vs 6.2%, p =0.02, see supplementary material, Table ST5). Overall, children with SEN status at KS2 were less likely to achieve level 4 or above in KS2 English, Maths and Science tests than those without formal support (52.6% vs 87.6%, p=0.00).

Educational Outcomes and SEN Status in Secondary School

Overall, almost three-quarters (72.1%) of the study sample achieved 5 or more GCSEs (including English and Maths) at A*-C grade, which is significantly higher than the national average of 47.6% children achieving this standard the same year(33). The proportion of children with hearing difficulties achieving this target was lower compared to those with normal hearing and vision (see Figure 2).

KS4: Achievement of ≥5 GCSEs at A*-C, including Maths & English

Confidential: For Review Only

	**Univariate logistic regression**		**Model 1 :** Male, SCBU admission, SEN at KS4		**Model 2:** Model 1 & maternal age <25, parity & smoking during pregnancy		**Model 3:** Model 2 & FAI & IMD		**Model 4:** Model 3 & KS2 English, Maths & Science performance		**Model 5:** Model 4 & IQ		**Model 6**: Model 5 & behaviour, social perceptions & attention	
	**OR**	**95% CI**	**OR**	**95% CI**	**OR**	**95% CI**	**OR**	**95% CI**	**OR**	**95% CI**	**OR**	**95% CI**	**OR**	**95% CI**
**Hearing difficulties** (n=852)	0.80	0.68 – 0.94	0.83	0.69 – 0.99	0.83	0.69 – 0.99	0.82	0.68 – 0.99	0.85	0.69 – 1.04	0.95	0.76 – 1.17	0.96	0.77 – 1.19
**Visual difficulties** (n=573)	0.86	0.71 – 1.05	0.94	0.74 – 1.17	1.00	0.79 – 1.27	1.02	0.81 – 1.30	1.20	0.92 – 1.56	1.34	1.00 – 1.79	1.37	1.02 – 1.84
**Co-occurring hearing & visual difficulties** (n=45)	0.55	0.29 – 1.06	0.67	0.29 – 1.51	0.68	0.29 – 1.58	0.66	0.28 – 1.53	1.13	0.43 – 2.94	1.42	0.50 – 4.06	1.49	0.52 – 4.29

Table 3: Multiple regression models using the imputed dataset (N=8,205) demonstrating the likelihood children achieved ≥5 GCSEs at A-C grade (including Maths & English), according to hearing and visual status and after adjustment for confounding variables (models 1-3) and mediating factors (models 4-5). OR=Odds Ratio*

Confidential: For Review Only

The association between hearing difficulties and poorer performance at GCSEs is attenuated after adjustment for performance at KS2 and IQ, suggesting that the association between hearing and education is mediated through these factors (see Table 3 for the imputed dataset analysis; see supplementary material, Table ST11 for the complete cases analysis). Children with visual difficulties alone were no less likely to attain 5 or more GCSEs at A*-C; they were in fact more likely to achieve this target after adjustment for confounding variables and IQ.

Children with co-occurring hearing and visual difficulties were less likely to achieve the national target even after adjustment for confounding factors, although this association was weak (OR 0.66, CI 0.28-1.53). This relationship is attenuated after adjustment for performance at KS2, suggesting that

poorer outcomes at KS4 in these children can be partly explained by poorer educational outcomes at primary school.

At KS4, the overall proportion of children receiving SEN support was similar to that at KS2 (6.7% vs 6.5%, p=0.76). Children with visual difficulties were almost twice as likely to receive SEN support than those with normal hearing and vision (see supplementary material, Table ST5). Children with SEN provision were less likely to attain 5 GCSEs at A*-C (32.3% vs 74.9%, p=0.00).

Discussion

Discussion of results

In this longitudinal study we have demonstrated that co-occurring mild hearing and visual difficulties in childhood have a negative impact on educational outcomes, greater than the effect of hearing or visual difficulties alone. We have shown a weak association between mild hearing difficulties at age 7 and academic achievement at KS2 and KS4, which may be mediated by IQ. We found no negative association between mild visual difficulties and academic outcomes. In contrast, children with co-occurring hearing and visual difficulties were less likely to attain the expected academic standards at the end of primary school, an effect which is not wholly mediated through IQ, behaviour, attention or social cognition, suggesting a substantial, unexplained educational disadvantage. They were also less likely to achieve 5 or more GCSEs at Key Stage 4, which may be attributable to poor performance at KS2. However, this relationship is weak, likely due to the relatively low prevalence of co-occurring mild sensory difficulties and potential small effect size.

A higher proportion of children with visual difficulties had SEN status at both primary school and secondary school relative to those with normal hearing and vision. Common visual difficulties, as defined by this study, are of themselves not sufficient to merit SEN support, suggesting a higher prevalence of additional disabilities amongst children with visual difficulties. This is consistent with

Confidential: For Review Only

previously reported findings(34). Children receiving SEN support are less likely to achieve the national targets at both primary school and secondary school compared to those without SEN support, implying SEN status is a proxy indicator of severity of educational difficulties.

Strengths and Weaknesses

The major strength of this study is that it uses a large population-based birth cohort, with an accurate assessment of hearing and vision. Furthermore, it uses standardised national tests as objective outcome measures, and longitudinally collected data on a wide range of confounders.

The most important limitation of this study relates to missing data and the under-representation of children from ethnic minorities and those from lower socio-economic backgrounds. A multiple imputation technique was utilised to minimise potential bias and improve precision. An additional limitation is the single time-point used to assess hearing, and possible inclusion of children with transient OME as a result, which may have led to underestimation of the effect of persistent OME-related hearing loss on academic attainment. Furthermore, we did not have information regarding which children were treated for their hearing and visual difficulties either prior or following the assessment at age 7. Finally, whilst we excluded children with known diagnoses of Down's Syndrome or Cerebral Palsy, we did not have full data regarding developmental disorders or medical co-morbidities. However, such conditions affect only a small minority of children with hearing or visual difficulties and this was a population-based study designed to evaluate the impact of common, mild difficulties(12, 35).

In the context of other research

Previous studies investigating the impact of co-occurring hearing and visual difficulties in childhood have focused on children with severe deficits. Developmental disorders commonly co-occur with severe hearing and visual difficulties, or 'deaf-blindness'(34-36), which often precludes children from attending mainstream schools and has additional implications for their education. To our knowledge,

Confidential: For Review Only

our study is the first to investigate the impact of co-occurring common mild hearing and visual difficulties upon academic attainment, in a population of children attending mainstream schools.

The impact of childhood OME on cognition and educational performance has been studied previously by prospective cohort studies, although there is no clear consensus. Data from the longitudinal Dunedin study demonstrated an association between childhood OME and lower IQ in early teenage years(2), which is consistent with findings from the Aarhus Birth Cohort that reported hearing loss at 9 -11 years was associated with behavioural problems and reading difficulties(8). In contrast, data from the Danish National Birth Cohort showed no association between childhood OME episodes and school performance, findings supported by a meta-analysis of prospective studies, which found little to no association between OME and speech and language development(6, 7). The disparity in these findings is likely explained by the heterogeneity in study design, including inconsistencies in adjustment for confounding variables and consideration of associated hearing loss and persistence of OME. Our findings demonstrated a small effect of OME and/or hearing loss at age 7 on academic performance at age 10-11 and 16 years after adjustment for confounding variables, mediated through IQ. This is consistent with data from the 1970 British Birth Cohort(3), and previously published work using ALSPAC data, which have shown an association between OME and lower IQ(4), although their results suggest this association diminishes with age.

Previous research investigating the impact of common visual difficulties in childhood on educational outcomes has focused largely on refractive errors, which were not included in this study(22, 23). There is, however, convincing evidence from a cross-sectional study involving participants of the Born in Bradford birth cohort study that reduced visual acuity at age 4-5 years is associated with reduced literacy(1). However, the prevalence of reduced visual acuity was considerably lower in our study (<1% vs 4%, likely due to the different socioeconomic and ethnic demographics of the cohorts). Data from the 1958 British Birth Cohort demonstrated no impact of unilateral amblyopia in childhood on educational tests at age 7, 11 and 16, or on highest educational qualification obtained(37).

Confidential: For Review Only

Amblyopia was the most common cause of visual difficulties in our study, and our findings are consistent with this.

Furthermore, we demonstrated higher rates of SEN amongst children with visual difficulties; this is consistent with previous research demonstrating that visual difficulties commonly co-occur with developmental disorders(35). We are, however, unable to explain the observed positive effect of visual difficulties on performance at secondary school after adjustment for confounders and IQ; a finding that has been reported in the context of refractive errors previously(22).

Clinical and Research Implications

This study has important clinical implications. Children with known visual or hearing difficulties should be routinely tested for additional sensory difficulties, as even mild co-occurring visual or hearing deficits are associated with poorer educational outcomes. The impact of co-occurring hearing and visual difficulties on performance at secondary school is largely explained by poor performance at primary school, hence early identification and intervention is essential.

Future research involving larger numbers of participants are required to replicate these findings and elucidate further the factors mediating this association. We recommend that future investigators focus next on the role of reading and language skills as potential mediators which could explain these findings.

Acknowledgements

We are extremely grateful to all the families who took part in this study, the midwives for their help in recruiting them, and the whole ALSPAC team, which includes interviewers, computer and laboratory technicians, clerical workers, research scientists, volunteers, managers, receptionists and nurses. The authors thank Dr Linda Hollen for providing advice on the statistical methodology.

Funding Statement

Confidential: For Review Only

The UK Medical Research Council, the Wellcome Trust (Grant ref: 102215/2/13/2) and the University of Bristol provide core support for ALSPAC. A comprehensive list of grants funding is available on the ALSPAC website: http://www.bristol.ac.uk/alspac/external/documents/grant-acknowledgements.pdf

Author Contributors

MH, AH, CW & AE designed the study. MH carried out the data analysis & wrote the initial manuscript draft. AH, CW & AE contributed to the analysis, and reviewed and revised the manuscript. The authors declare that there is no conflict of interest in the publication of this research. This publication is the work of the authors, who will serve as guarantors for the contents of this paper.

References

1. Bruce A, Fairley L, Chambers B, Wright J, Sheldon TA. Impact of visual acuity on developing literacy at age 4-5 years: a cohort-nested cross-sectional study. BMJ Open. 2016;6(2):e010434.
2. Bennett KE, Haggard MP, Silva PA, Stewart IA. Behaviour and developmental effects of otitis media with effusion into the teens. Archives of disease in childhood. 2001;85(2):91-5.
3. Bennett KE, Haggard MP. Behaviour and cognitive outcomes from middle ear disease. Archives of disease in childhood. 1999;80(1):28-35.
4. Hall AJ, Maw R, Midgley E, Golding J, Steer C. Glue ear, hearing loss and IQ: an association moderated by the child's home environment. PloS one. 2014;9(2):e87021.
5. Archbold S YZ, Harrigan S Gregory S, Wakefield t, Holland L, Mulla I. Experiences of young people with mild to moderate hearing loss. National Deaf Children's Society; 2015.
6. Fougner V, Korvel-Hanquist A, Koch A, Dammeyer J, Niclasen J, Lous J, et al. Early childhood otitis media and later school performance - A prospective cohort study of associations. Int J Pediatr Otorhinolaryngol. 2017;94:87-94.
7. Roberts JE, Rosenfeld RM, Zeisel SA. Otitis media and speech and language: a meta-analysis of prospective studies. Pediatrics. 2004;113(3 Pt 1):e238-48.
8. Niclasen J, Obel C, Guldager C, Pleinert S, Dammeyer J. Associations between childhood hearing loss and behavioural and academic difficulties: A Danish cohort study. Int J Pediatr Otorhinolaryngol. 2016;90:91-8.
9. Carlton J, Karnon J, Czoski-Murray C, Smith KJ, Marr J. The clinical effectiveness and cost-effectiveness of screening programmes for amblyopia and strabismus in children up to

Confidential: For Review Only

the age of 4-5 years: a systematic review and economic evaluation. Health technology assessment (Winchester, England). 2008;12(25):iii, xi-194.
10. Williams C, Northstone K, Howard M, Harvey I, Harrad RA, Sparrow JM. Prevalence and risk factors for common vision problems in children: data from the ALSPAC study. Br J Ophthalmol. 2008;92(7):959-64.
11. Robaei D, Kifley A, Gole GA, Mitchell P. The impact of modest prematurity on visual function at age 6 years: findings from a population-based study. Archives of ophthalmology (Chicago, Ill : 1960). 2006;124(6):871-7.
12. Pathai S, Cumberland PM, Rahi JS. Prevalence of and early-life influences on childhood strabismus: findings from the Millennium Cohort Study. Archives of pediatrics & adolescent medicine. 2010;164(3):250-7.
13. Erdener D, Burnham D. The relationship between auditory-visual speech perception and language-specific speech perception at the onset of reading instruction in English-speaking children. Journal of experimental child psychology. 2013;116(2):120-38.
14. McGurk H, MacDonald J. Hearing lips and seeing voices. Nature. 1976;264(5588):746-8.
15. Boyd A, Golding J, Macleod J, Lawlor DA, Fraser A, Henderson J, et al. Cohort Profile: the 'children of the 90s'--the index offspring of the Avon Longitudinal Study of Parents and Children. Int J Epidemiol. 2013;42(1):111-27.
16. Fraser A M-WC, Tilling K, Boyd A, Golding J, Davey Smith G, Henderson J, Macleod J,, Molloy L NA, Ring S, Nelson SM, Lawlor DA. Cohort Profile: The Avon Longitudinal Study of Parents and Children: ALSPAC mothers cohort. International Journal of Epidemiology. 2013;42:97-110.
17. Recommended Procedure : Pure-tone air-conduction and bone-conduction threshold audimetry with and without masking. The British Society of Audiology; 2011.
18. Midgley EJ, Dewey C, Pryce K, Maw AR. The frequency of otitis media with effusion in British pre-school children: a guide for treatment. ALSPAC Study Team. Clinical otolaryngology and allied sciences. 2000;25(6):485-91.
19. Daly KA, Hunter LL, Giebink GS. Chronic otitis media with effusion. Pediatrics in review. 1999;20(3):85-93.
20. Bluestone CD. Otitis Media in Infants and Children. 4th ed: People's Medical Publishing House--USA LTD; 2006.
21. ICD-11 Internation Classification of Disease for Mortality and Morbidity Statistics. World Health Organisation; 2018.
22. Saw SM, Tan SB, Fung D, Chia KS, Koh D, Tan DT, et al. IQ and the association with myopia in children. Invest Ophthalmol Vis Sci. 2004;45(9):2943-8.
23. Mutti DO, Mitchell GL, Moeschberger ML, Jones LA, Zadnik K. Parental myopia, near work, school achievement, and children's refractive error. Invest Ophthalmol Vis Sci. 2002;43(12):3633-40.
24. Education TDf. National Pupil Database, UK data archive: Colchester, Essex.
25. Benton T ST. Analysis of use of Key Stage 2 data in GCSE predictions. ARD Research Division; 2014.
26. Paradise JL, Rockette HE, Colborn DK, Bernard BS, Smith CG, Kurs-Lasky M, et al. Otitis media in 2253 Pittsburgh-area infants: prevalence and risk factors during the first two years of life. Pediatrics. 1997;99(3):318-33.

Confidential: For Review Only

27. Cumberland PM, Pathai S, Rahi JS, Millennium Cohort Study Child Health G. Prevalence of eye disease in early childhood and associated factors: findings from the millennium cohort study. Ophthalmology. 2010;117(11):2184-90 e1-3.
28. Sirin SR. Socioeconomic Status and Academic Achievement: A Meta-Analytic Review of Research. Review of Educational Research. 2005;75(3):417-53.
29. Bowatte G, Tham R, Allen KJ, Tan DJ, Lau M, Dai X, et al. Breastfeeding and childhood acute otitis media: a systematic review and meta-analysis. Acta Paediatr. 2015;104(467):85-95.
30. Baron RM, & Kenny, D. A. The moderator–mediator variable distinction in social psychological research: Conceptual, strategic, and statistical considerations. Journal of Personality and Social Psychology. 1986;51(6):1173-82.
31. Sterne JA, White IR, Carlin JB, Spratt M, Royston P, Kenward MG, et al. Multiple imputation for missing data in epidemiological and clinical research: potential and pitfalls. Bmj. 2009;338:b2393.
32. National Curriculum Assessments at Key Stage 2 in England 2008 (Revised). England: Department for Children, Schools andFfamilies; 2009.
33. GCSE and Equivalent Examination Results in England 2007/08 (Revised). Department for Children, Schools and Families; 2009.
34. Salt A, Sargent J. Common visual problems in children with disability. Archives of disease in childhood. 2014;99(12):1163-8.
35. Kancherla V, Van Naarden Braun K, Yeargin-Allsopp M. Childhood vision impairment, hearing loss and co-occurring autism spectrum disorder. Disabil Health J. 2013;6(4):333-42.
36. NDCS. Prevalence of additional disabilities with deafness: A review of the literature. 2012.
37. Rahi JS, Cumberland PM, Peckham CS. Does amblyopia affect educational, health, and social outcomes? Findings from 1958 British birth cohort. BMJ. 2006;332(7545):820-5.

Figure Legends

Figure 1: Study sample flow chart showing inclusion & exclusion criteria

Figure 2: Graph showing proportion of students achieving ≥5 GCSEs at A-C (including Maths & English) by hearing and vision status*

Confidential: For Review Only

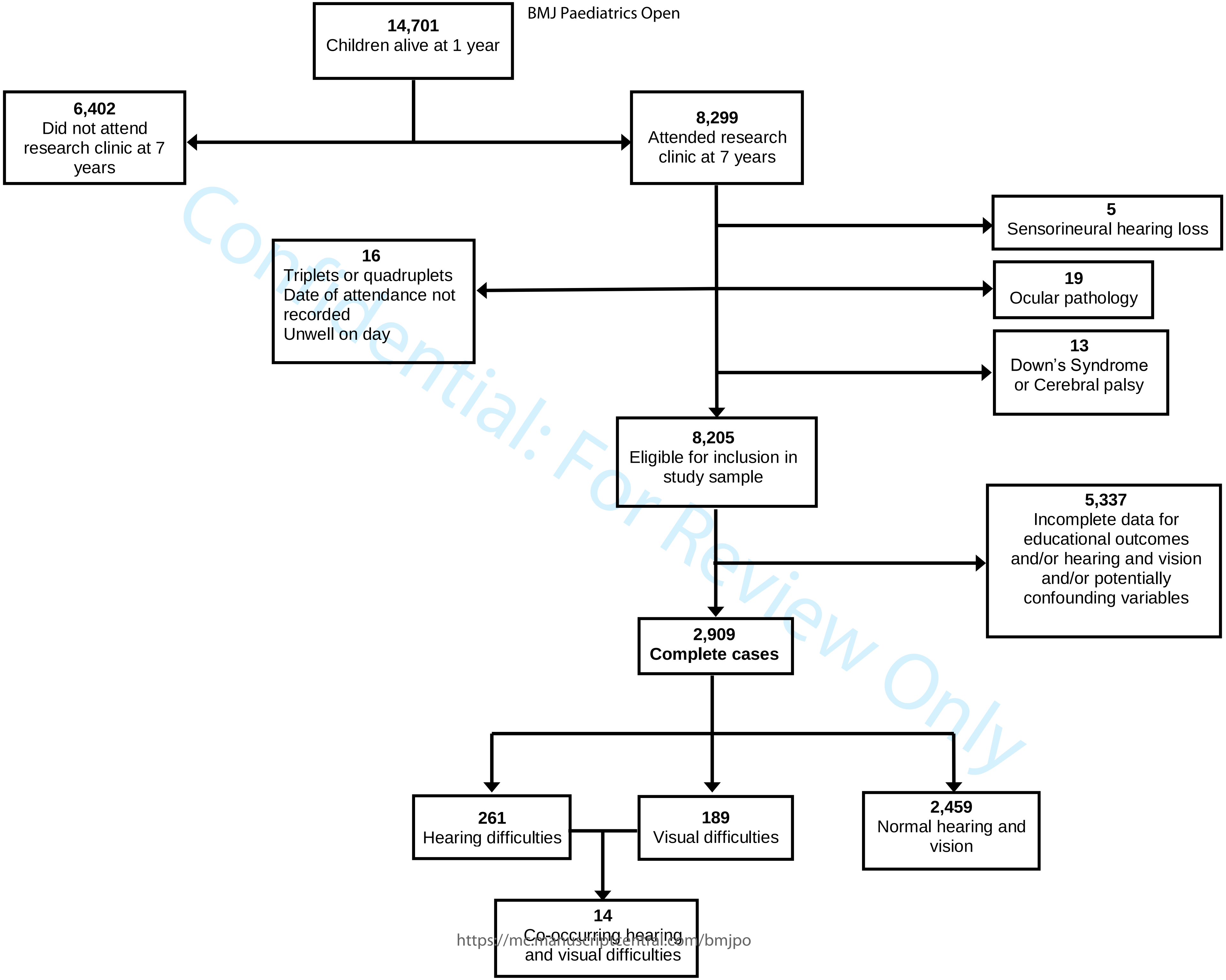

Confidential: For Review Only

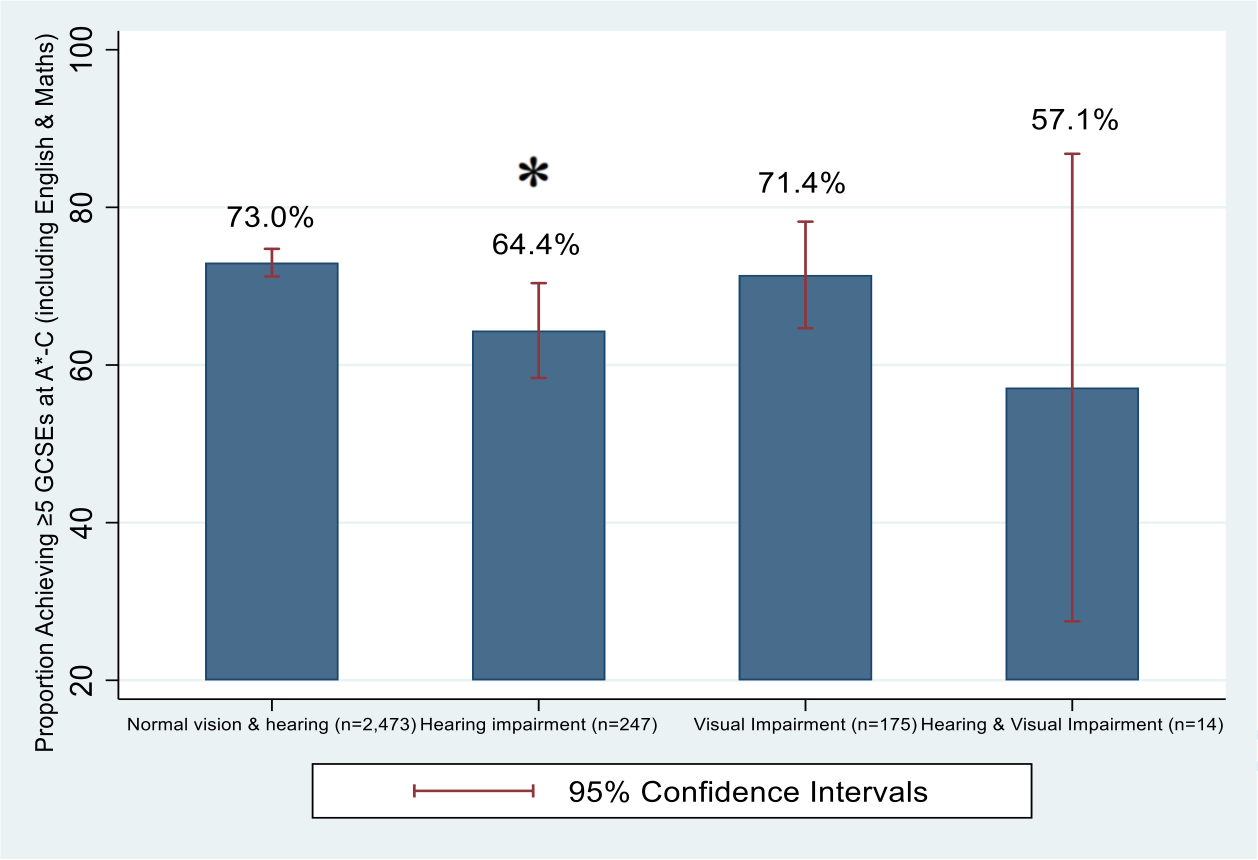
Proportion Achieving ≥5 GCSEs at A*-C (including English & Maths)
20
40
60
80
100
73.0%
64.4%
*
71.4%
57.1%
Normal vision & hearing (n=2,473)
Hearing impairment (n=247)
Visual Impairment (n=175)
Hearing & Visual Impairment (n=14)
95% Confidence Intervals
